# Over-expression of *AURKA*, *SKA3* and *DSN1* contributes to colorectal adenoma to carcinoma progression

**DOI:** 10.18632/oncotarget.9960

**Published:** 2016-06-13

**Authors:** Tzu-Po Chuang, Jaw-Yuan Wang, Shu-Wen Jao, Chang-Chieh Wu, Jiann-Hwa Chen, Koung-Hung Hsiao, Chung-Yen Lin, Shu-Hwa Chen, Sheng-Yao Su, Ying-Ju Chen, Yuan-Tsong Chen, Deng-Chyang Wu, Ling-Hui Li

**Affiliations:** ^1^ Taiwan International Graduate Program in Molecular Medicine, National Yang-Ming University and Academia Sinica, Taipei, Taiwan; ^2^ Institute of Biochemistry and Molecular Biology, National Yang-Ming University, Taipei, Taiwan; ^3^ Division of Gastroenterology and General Surgery, Department of Surgery, Kaohsiung Medical University Hospital, Kaohsiung Medical University, Kaohsiung, Taiwan; ^4^ Graduate Institute of Clinical Medicine, College of Medicine, Kaohsiung Medical University, Kaohsiung, Taiwan; ^5^ Center for Biomarkers and Biotech Drugs, Kaohsiung Medical University, Kaohsiung, Taiwan; ^6^ Department of Surgery, Division of Colon and Rectal Surgery, Tri-Service General Hospital, National Defense Medical Center, Taipei, Taiwan; ^7^ Department of Internal Medicine, Tzu Chi General Hospital, Taipei Branch, Taipei, Taiwan; ^8^ Department of Colorectal Surgery, Tzu Chi General Hospital, Taipei Branch, Taipei, Taiwan; ^9^ Institute of Information Science, Academia Sinica, Taipei, Taiwan; ^10^ Institute of Biomedical Sciences, Academia Sinica, Taipei, Taiwan; ^11^ Division of Gastroenterology, Department of Internal Medicine, Kaohsiung Medical University Hospital, Kaohsiung, Taiwan; ^12^ Department of Internal Medicine, Kaohsiung Municipal Ta-Tung Hospital, Kaohsiung, Taiwan; ^13^ Center for Infectious Disease and Cancer Research, Kaohsiung Medical University, Kaohsiung, Taiwan

**Keywords:** colon cancer progression, malignant transformation, AURKA, SKA3, DSN1

## Abstract

Development of colorectal cancer (CRC) involves sequential transformation of normal mucosal tissues into benign adenomas and then adenomas into malignant tumors. The identification of genes crucial for malignant transformation in colorectal adenomas (CRAs) has been based primarily on cross-sectional observations. In this study, we identified relevant genes using autologous samples. By performing genome-wide SNP genotyping and RNA sequencing analysis of adenocarcinomas, adenomatous polyps, and non-neoplastic colon tissues (referred as tri-part samples) from individual patients, we identified 68 genes with differential copy number alterations and progressively dysregulated expression. Aurora A, SKA3, and DSN1 protein levels were sequentially up-regulated in the samples, and this overexpression was associated with chromosome instability (CIN). Knockdown of SKA3 in CRC cells dramatically reduced cell growth rates and increased apoptosis. Depletion of SKA3 or DSN1 induced G2/M arrest and decreased migration, invasion, and anchorage-independent growth. *AURKA* and *DSN1* are thus critical for chromosome 20q amplification-associated malignant transformation in CRA. Moreover, *SKA3* at chromosome 13q was identified as a novel gene involved in promoting malignant transformation. Evaluating the expression of these genes may help identify patients with progressive adenomas, helping to improve treatment.

## INTRODUCTION

Colorectal cancer (CRC) is one of the leading causes of cancer mortality worldwide [1]. Five-year survival rates for early-stage CRC patients (TNM stages I-II) range from 70-90%, but drop dramatically to 10-60% for advanced-stage patients (TNM stages III-IV) [2, 3], demonstrating the importance of early detection. Multiple genetic changes, including *APC*, *KRAS*, and *TP53* mutations, and the loss of SMAD2 and SMAD4 activities promote CRC development [4–6]. This classic model outlined by Fearon and Vogelstein applies to 60% of sporadic CRC cases [6]. Although colorectal adenoma (CRA) is the precursor of CRC, the majority of CRAs do not progress into CRCs; only 5% of adenomas are estimated to develop into carcinomas [7, 8]. This implies the existence of a barrier of malignant transformation and crucial molecular alterations may trigger progression from CRA to CRC.

Chromosomal instability (CIN), microsatellite instability (MSI), and CpG island methylator phenotype contribute to malignant transformation [9–11]. CIN, which is present in about 85% of CRC patients, increases the adaptability of tumor cells to the tumor microenvironment [10, 12]. CIN also alters gene expression, which is critical for carcinogenesis. The most frequently reported chromosomal alterations in CRC are gains of 7p, 7q, 8q, 13q, and 20q and losses of 8p, 15q, 17p, and 18q [13–17]. Among these, 20q amplification is believed to promote the overexpression of *AURKA* (20q13.2) and *TPX2* (20q11), ultimately promoting progression from CRA to CRC [18]. However, the underlying mechanisms of CIN are still largely unknown.

Early detection and intervention in CRC patients and adenoma patients with a high risk of malignant transformation are effective in reducing cancer-associated mortality [19]. To improve early detection, a better understanding of biological mechanisms driving adenoma-carcinoma progression is necessary. The current genetic model of CRC progression is mostly based on cross-sectional studies, which compare adenomas and carcinomas from different individuals [20–26]. While such studies are certainly valuable, they do not provide detailed information about progression in individual lesions. However, longitudinal studies of CRC progression are very difficult to conduct due to ethical issues. Here, we attempted to identify critical genes involved in CRA to CRC progression within individual patients. Integrative genomic analysis was performed in carcinoma, paired adenomatous polyp, and paired non-neoplastic colon tissues (referred as tri-part samples) from the same patient. Among the identified candidate genes, *AURKA*, *SKA3*, and *DSN1*, which are involved in cell cycle transition, cell growth, and proliferation, were progressively up-regulated in paired non-neoplastic colon tissues, adenomas, and carcinomas. Our study thus identified specific genes that contribute to CIN-driven progression from CRA to CRC.

## RESULTS

### Genomic instability in tumor samples

The tissue samples from 76 CRC patients were subjected to a microsatellite instability (MSI) assay. Genomic DNA was not available from seven small polyp tissues, which were subjected to protein isolation but not the MSI assay. Eight out of the 76 carcinomas were classified as MSI-H, 5 as MSI-L, and 63 as MSS (Supplementary Table S1); one of the 69 polyps was classified as MSI-H, 2 as MSI-L, and 66 as MSS. These results are consistent with the literature reporting that 10-15% of sporadic CRCs are MSI cancers [27].

**Figure 1:**
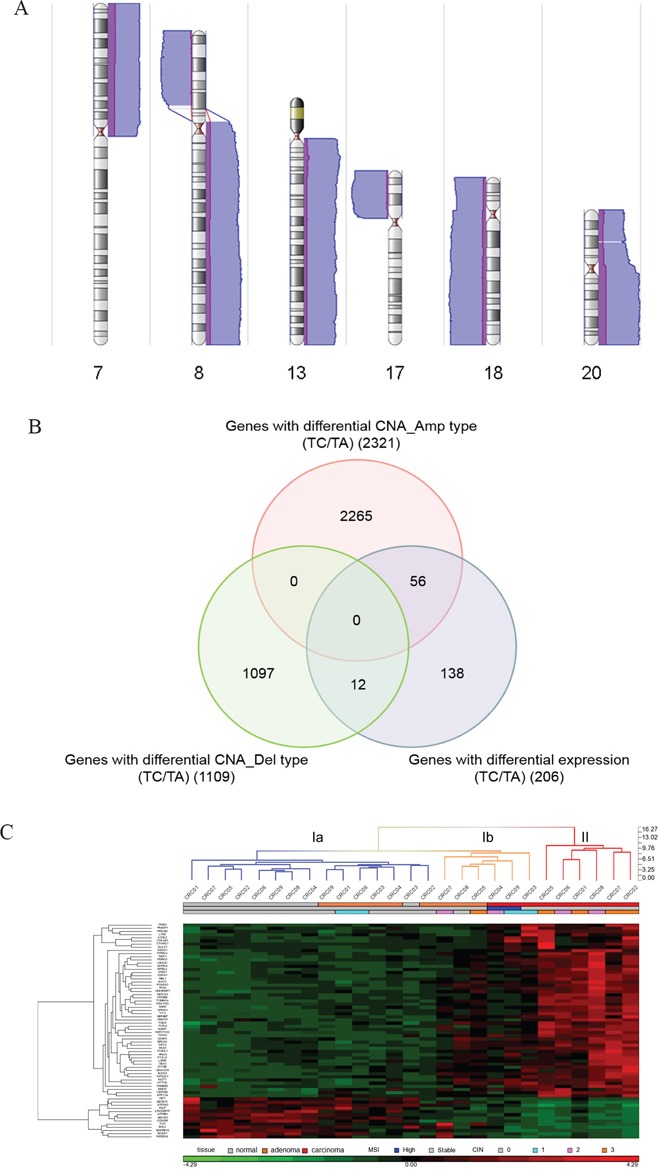
Identification of malignant transformation-related genes **A.** Genomic regions with differential copy number alterations in carcinoma samples compared to polyp samples. Purple bars indicate CNAs detected in polyp samples and blue bars indicate CNAs detected in carcinoma samples. Bars on the right side represent amplification-type CNAs while bars on the left side represent deletion-type CNAs. The heights of the bars indicate the numbers of samples with CNAs in that region. Chromosome numbers are indicated at the bottom of each karyogram. **B.** Venn diagram of genes with differential expression and genes with differential CNAs in carcinoma samples. The number of genes in each list is shown in parentheses. **C.** Hierarchical clustering of the tissue samples and the 68 candidate genes. The tissue samples include nine carcinomas, nine paired polyps, and nine paired non-neoplastic colon tissues. mRNA expression levels are shifted to a mean of zero and scaled to a deviation of one, with green representing the lowest level and red representing the highest level. The tissue group, MSI status, and CIN level of each sample is indicated under the sample column. Blue dendrogram columns belong to cluster Ia, orange to cluster Ib, and red to cluster II.

The same sample set, except for two polyps, was subjected to chromosomal aberration detection with an Affymetrix Genome-wide SNP6.0 Array. Somatic copy number alterations (CNA) were identified by comparing tumor samples to paired normal tissues. Global CNAs in polyp and adenocarcinoma samples are presented in Supplementary Figure S1 and Supplementary Table S1. To identify chromosomal regions that were differentially altered during tumor progression, CNA regions identified in polyps and carcinoma samples were compared using an analysis of variance (ANOVA). Amplifications at chromosomes 7p, 8q, 13q, 20p, and 20q and deletions at chromosomes 8p, 17p, 18p and 18q were increased in carcinomas compared to polyps (Figure 1A). Well-known tumor suppressor genes, like *TP53* on chromosome 17p and *SMAD2* and *SMAD4* on chromosome 18q, were deleted more frequently in carcinomas than in polyps. In contrast, deletion of the *APC* locus on chromosome 5q did not significantly differ in carcinomas compared to polyps. These results are consistent with evidence that loss of *APC*, which is common in adenomagenesis and carcinomagenesis, occurs very early in CRC tumorigenesis, while loss of *TP53*, *SMAD2*, and *SMAD4* occurs later. Therefore, our findings are consistent with previous reports and may help identify new malignant transformation-related genes.

### Genes with differential expression during tumor progression

To identify genes for which deregulation correlated with CNA during tumor progression and might be critical for malignant transformation, we performed global expression profiling of the tri-part samples from nine patients using RNA-seq. A total of 609 genes for which expression was progressively dysregulated as normal mucosa tissue developed into polyps and eventually carcinoma were identified by K-mean clustering. Among them, 206 were also deregulated more frequently in carcinomas compared to polyps and adjacent normal tissues (Supplementary Figure S2A). Pathway analysis of these 206 genes revealed that they were enriched in cell survival, cell proliferation, and cell cycle, particularly at the G2/M phase (Supplementary Figure S2B and Supplementary Table S2).

Among the 206 genes, 12 were located on differentially deleted chromosomal regions and 56 were on differentially amplified regions in carcinoma samples (Figure 1B and Supplementary Table S3). Pathway analysis of these 68 genes revealed that the three top molecular and cellular functions in which they were involved were cell cycle, cell death and survival, and cellular movement (Supplementary Figure S3). Hierarchical cluster analysis of the 27 tissue samples revealed that the majority of carcinoma samples had distinct expression profiles for these 68 genes compared to adjacent normal tissues and polyp samples (Figure 1C). Interestingly, CRC04 and CRC09 carcinomas with MSI-H, CRC03 carcinoma with low-degree CIN, CRC05 polyp with high-degree CIN, CRC07 polyp with carcinoma lesions, and CRC08 polyp with severe dysplasia formed a sub-cluster Ib that was distinct from the other normal and polyp samples. These results strongly suggested that the deregulation of these 68 genes might contribute to malignant transformation, and the expression profile of these genes identified polyp samples with high risks of transformation.

### Protein expression levels of candidate genes during tumor progression

**Figure 2:**
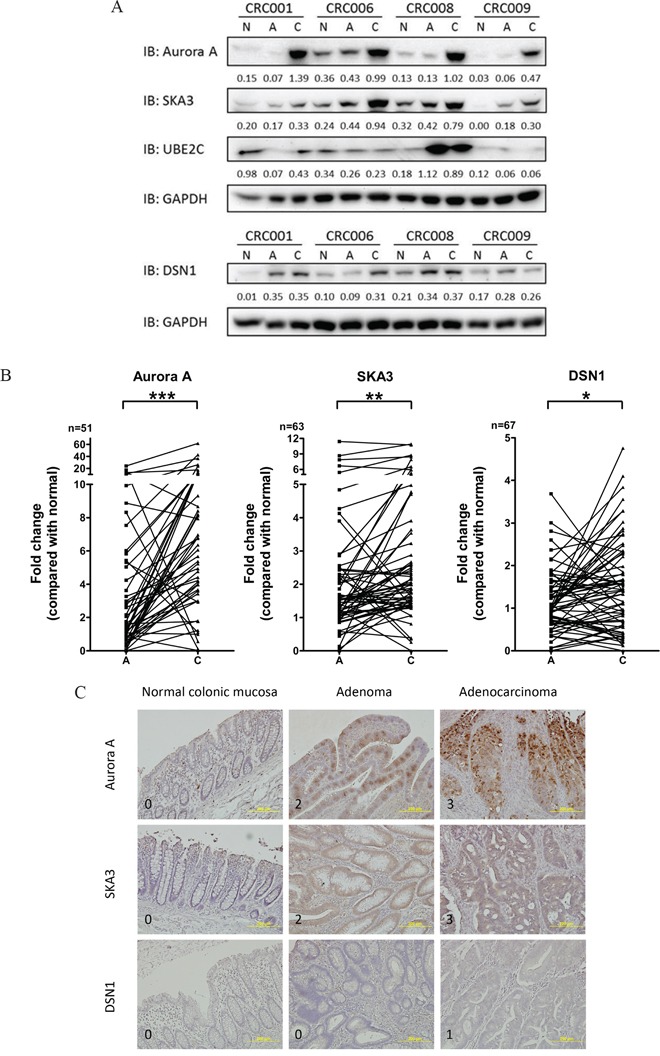
Protein expression of candidate genes in tissues **A.** Representative immunoblotting analysis of Aurora A, SKA3, UBE2C, and DSN1 in tri-part samples from four patients (CRC01, CRC06, CRC08, and CRC09). Ratios normalized to GAPDH are shown under each lane. (N, non-neoplastic tissue; A, polyp; C, carcinoma) **B.** Aurora A, SKA3, and DSN1 protein levels in paired adenoma and carcinoma samples. Fold change in protein levels was normalized to paired non-neoplastic samples. (*p<0.01; **p<0.001; ***p<0.0001) **C.** Representative images of IHC staining of Aurora A, SKA3, and DSN1 in tri-part samples are shown. Each row of images shows the tri-part samples from a single patient. Signal intensity scores are indicated on the bottom left of each image. Magnification: 200X.

Among the 68 candidate genes, those that were over-expressed might be the most useful as therapeutic targets and/or diagnosis biomarkers. In addition, since CIN is an important feature associated with malignant transformation, we focused on the up-regulated genes that are reportedly involved in chromosome segregation and/or aneuploidy. The *AURKA*, *UBE2C*, and *DSN1* genes at chromosome 20q and the *SKA3* gene at chromosome 13q were selected for validation at the protein level using an immunoblotting assay. Progressive increases in Aurora A, SKA3, and DSN1 protein levels were observed in the initially screened set of tri-part samples (Figure 2A). Protein levels and mRNA expression were positively correlated for *AURKA*, *SKA3*, and *DSN1* and negatively correlated for *UBE2C* (Table 1). Therefore, we further examined Aurora A, SKA3, and DSN1 protein levels in the remaining 67 CRC patients and a second cohort of 30 patients in an immunoblotting assay. Because the gene expression profiles in carcinomas with and without MSI were different (Figure 1C), patients with MSI in either carcinoma or polyp tissues were excluded from the statistical analysis. We also excluded patients with adenocarcinoma nodules detected in polyp tissues, those with extreme changes in protein levels, and those without detectable protein levels in the tri-part tissues from further analysis. We confirmed that Aurora A, SKA3, and DSN1 proteins were up-regulated in the adenoma and carcinoma tissues (Figure 2B). Immunohistochemistry (IHC) staining of formalin-fixed and paraffin-embedded (FFPE) tissue sections from a subset of the patients was also used to confirm these findings; the results were consistent with the immunoblotting assay (Figure 2C and Supplementary Table S4).

**Table 1:** Correlation of RNA and protein expression in 9 patients

Gene name	Physical position	Upregulation in CRC patients^#^		Pearson correlation coefficient
		mRNA	protein	
*AURKA*	Chr20:54944445-54967351	Yes	Yes	0.629
*SKA3*	Chr13:21727734-21750741	Yes	Yes	0.603
*DSN1*	Chr20:35380194-35402230	Yes	Yes	0.450
*UBE2C*	Chr20:44441255-44445596	Yes	No	−0.321

# Upregulation is defined as that more than 50% of tumor tissue samples consisting of polyps and carcinomas have a fold change ≥ 1.5 when compared to paired non-neoplastic tissues.

**Figure 3:**
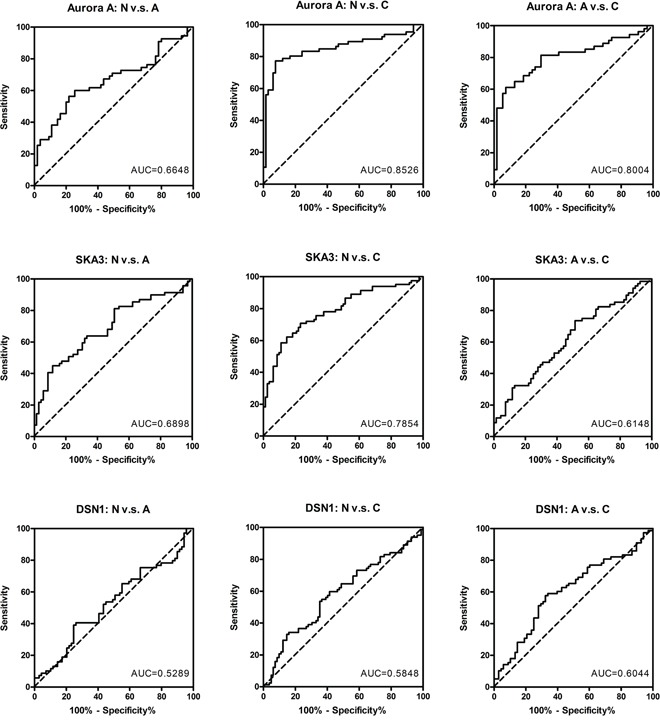
ROC analysis of Aurora A, SKA3, and DSN1 for discriminating carcinoma from adenoma ROC curves show the specificity and sensitivity of each protein by comparing polyps to non-neoplastic tissues (left panel), carcinomas to non-neoplastic tissues (middle panel), and carcinomas to polyps (right panel). The AUC values are indicated in each graph.

The potential of each gene to discriminate tissue types was determined using receiver-operator characteristic (ROC) curves (Figure 3). Aurora A showed the highest accuracy in discriminating carcinoma from polyp (AUC = 0.8004) and carcinoma from normal tissue (AUC = 0.8526), and SKA3 showed the highest sensitivity and specificity in discriminating polyp from normal tissue (AUC = 0.6898). The moderate accuracy of SKA3 and DSN1 in discriminating carcinoma from polyp might be due to the inclusion of high-risk adenomas in the polyp groups.

### Correlations between protein levels and clinicopathological features and genomic alterations

Next, we determined correlations between protein levels in adenoma and carcinoma samples and clinicopathological features. For adenoma samples, increased protein levels were not associated with either grade of dysplasia or polyp size (Supplementary Table S5), suggesting that increased levels of the candidate proteins and severe dysplasia or larger polyp sizes may be independent predictors of a high risk of malignant transformation. Protein levels in carcinoma tissues were also not associated with the following clinicopathological features: gender, age at diagnosis, carcinoma location, carcinoma size, clinical TNM stage, stage of primary tumor (T), and lymph node involvement (N) (Supplementary Table S6).

**Figure 4:**
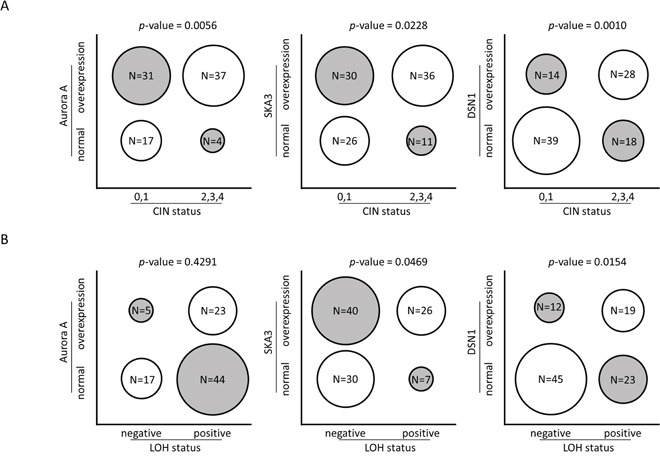
Correlations between protein levels and genomic alteration Each circle represents the proportion of tumor samples in each category. Fold change of protein levels in tumor samples were normalized to paired non-neoplastic tissues, and overexpression is defined as fold change ≥ 1.5. **A.** Overexpression of Aurora A, SKA3, and DSN1 protein is positively correlated with higher CIN (0 corresponds to CIN-stable, 1 to low degree of CIN, 2 to medium degree of CIN, 3 to high degree of CIN, and 4 to ultra-high degree of CIN). **B.** Overexpression of SKA3 and DSN1 proteins are correlated with the presence of LOH.

We further examined associations between protein levels in polyp and carcinoma samples and genomic and molecular alterations. The level of a candidate protein was treated as a dichotomous variable (Fold-change ≥ 1.5 vs. Fold-change < 1.5) and Fisher's exact test was performed. Increased Aurora A, SKA3, and DSN1 levels were associated with higher CIN status (Figure 4A, Supplementary Table S7). Higher SKA3 and DSN1 levels were also associated with loss of heterozygosity (LOH) (Figure 4B, Supplementary Table S7). Moreover, increased Aurora A and DSN1 levels were associated with chromosome 20q amplification. However, higher SKA3 levels were not associated with chromosome 13q amplification. We further examined whether gene amplification rather than whole chromosome arm duplication was associated with SKA3 overexpression. The gene dosage of SKA3 as determined by genomic real-time quantitative PCR was not correlated with protein levels (P value = 0.2546). Many more tumor tissues had high SKA3 levels than had 13q and SKA3 gene amplification (Supplementary Table S7). These observations suggest that DNA amplification plays a major role in the overexpression of the *AURKA* and *DSN1* genes, while mechanisms other than 13q and *SKA3* gene amplification are involved in deregulation of the *SKA3* gene. Intriguingly, higher SKA3 levels were associated with higher Aurora A (P value = 0.0305) and DSN1 (P value = 0.0021) levels (Supplementary Table S7). These results reveal that increases in Aurora A, SKA3, and DSN1 levels are not only associated with high CIN status but are also highly associated with each other. The interplay among these proteins may be an important contributor to malignant transformation in colorectal adenoma.

### Depletion of SKA3 reduced cell growth rate and induced apoptosis in CRC cells

**Figure 5:**
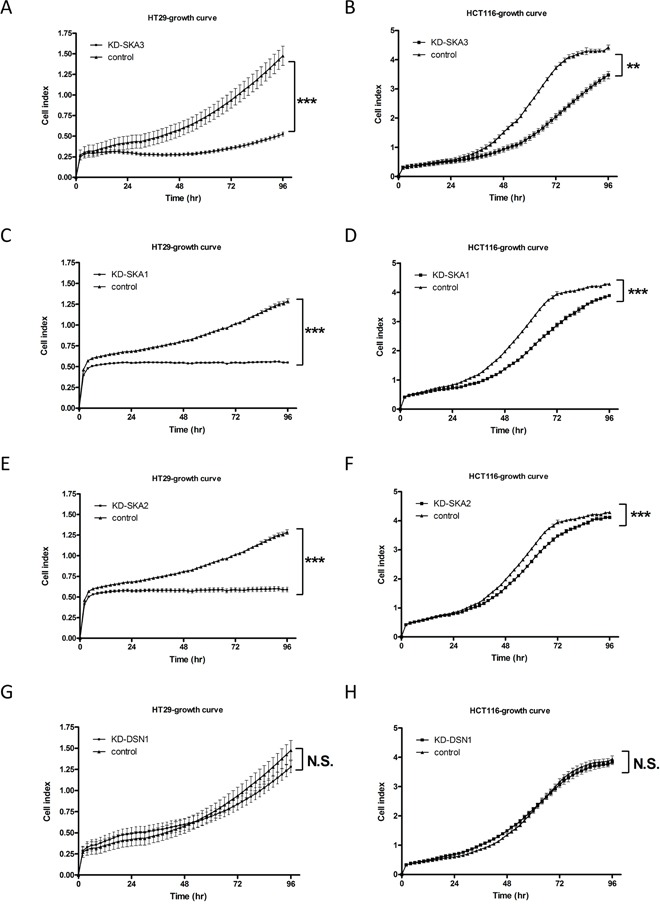
Knockdown of SKA3 reduces cell growth HT29 and HCT116 cells were transfected with control siRNA or target siRNA. A typical result is shown; each data point represents mean ± SD of cell indexes conducted in triplicate. **A-F.** Knockdown of SKA3, SKA1, or SKA2 reduced growth rates in HT29 and HCT116 cells. **G-H.** Knockdown of DSN1 did not affect cell growth rate in either HT29 or HCT116 cells. (**p<0.01; ***p<0.001; N.S., not significant).

The tumorigenic ability of the *AURKA* gene has been reported in CRC cell lines [18]. Therefore, we focused on elucidating the pathogenic roles of SKA3 and DSN1 in malignant transformation. We knocked down SKA3 or DSN1 expression using siRNA in HT29 (CIN-high) and HCT116 (CIN-stable) CRC cell lines. SKA3 protein was overexpressed in both cell lines while DSN1 was only overexpressed in HCT116 cells (Supplementary Figure S4A). The reduction of target gene expression by siRNA was confirmed by qPCR as well as an immunoblotting assay (Supplementary Figure S4B and S4C). Depletion of SKA3 reduced the cell growth rate in both cell lines (Figure 5A and 5B), and this inhibition was stronger in HT29 than in HCT116 cells despite comparable knockdown efficiency (Supplementary Figure S4C). SKA3 knockdown also increased apoptosis in both cell lines (Supplementary Figure S5).

Previous reports demonstrated that SKA3 forms a functional complex with SKA1 and SKA2 to promote mitotic exit in HeLa cells [28, 29]. To investigate whether the disruption of cell growth by depletion of SKA3 was caused by the loss of function of the SKA complex or SKA3 itself, we depleted SKA1 or SKA2 in HT29 and HCT116 cells. Knockdown of SKA1 or SKA2 reduced cell growth similarly to SKA3 knockdown (Figure 5C-5F). In contrast, depletion of DSN1 affected neither growth rate nor apoptosis in either cell line (Figure 5G and 5H; Supplementary Figure S5).

### SKA3 and DSN1 affected cell cycle progression in CRC cells

**Figure 6:**
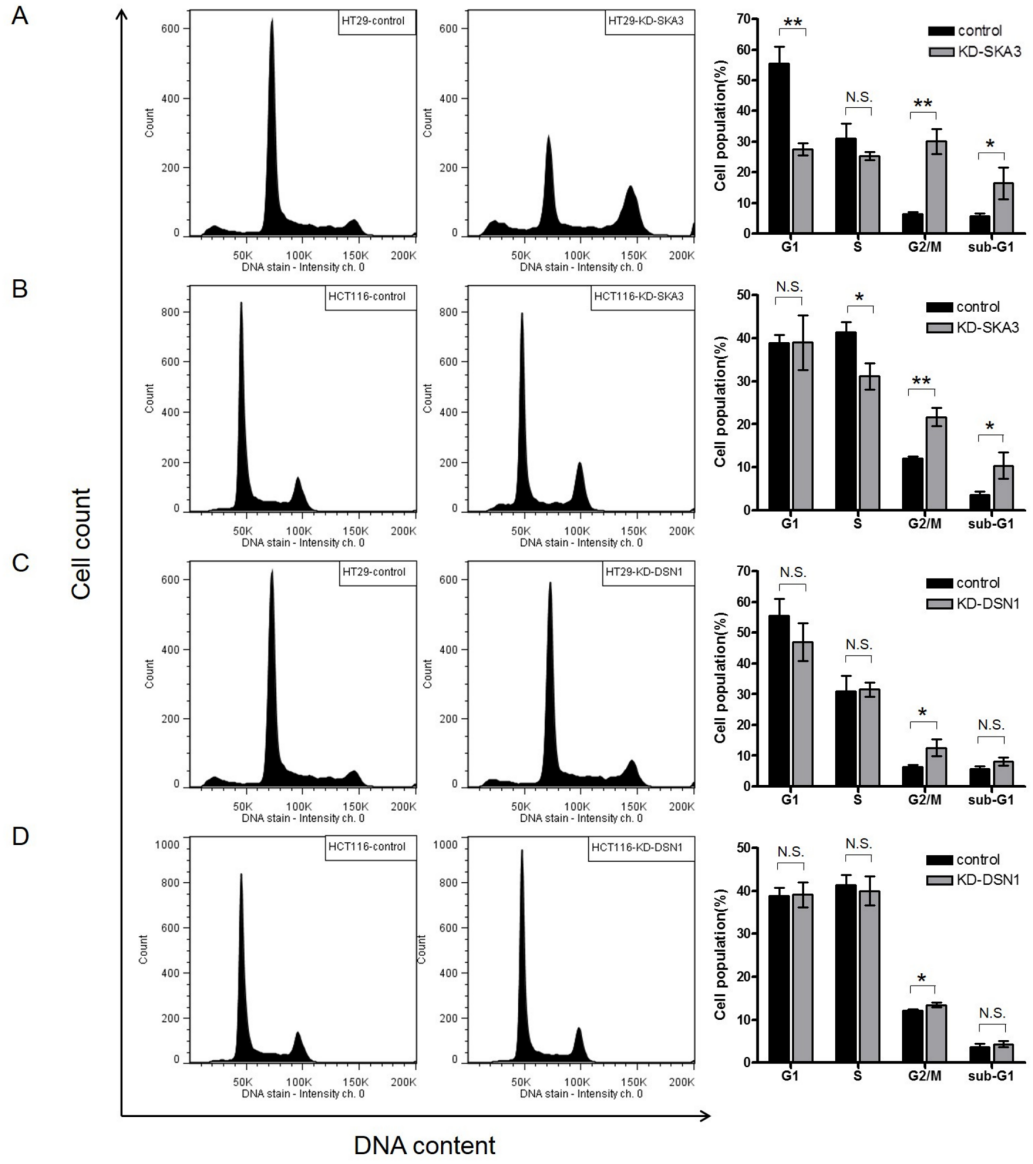
Knockdown of SKA3 or DSN1 inhibits cell cycle progression HT29 and HCT116 cells were transfected with control siRNA or target siRNA. A typical result of cell cycle distribution analysis is presented, with control siRNA in the left column and target RNA in the middle column. Quantification of the results of three independent experiments is presented in the right column. The percentages of cells at each cell cycle phase are shown as mean ± SD. **A.** SKA3 knockdown decreased the G1 phase population, increased the G2/M population, and increased the sub-G1 population in HT29 cells. **B.** SKA3 knockdown decreased the S phase population, increased the G2/M population, and increased the sub-G1 population in HCT116 cells. Knockdown of DSN1 increased G2/M arrest in **C.** HT29 cells and **D.** HCT116 cells. (*p<0.05; **p<0.01; N.S., not significant).

To further characterize how depletion of SKA3 affected cell growth, we performed cell cycle analysis. Knockdown of SKA3 strongly increased G2/M phase arrest and increased the sub-G1 population in both cell lines (Figure 6A and 6B). Again, the effect of SKA3 depletion was much stronger in HT29 than in HCT116 cells. To investigate possible off-target effects on cell cycle progression resulting from SKA3 knockdown, four individual siRNAs targeting SKA3 were independently transfected and assayed. Similar results were observed for all four siRNA oligos, suggesting that the effect of SKA3 knockdown on cell cycle progression was not due to off-target effects (Supplementary Figure S6). Knockdown of SKA1 or SKA2 affected cell cycle distribution similarly to SKA3 knockdown (Supplementary Figure S7). These results strongly suggest that the SKA3 knockdown-induced disturbance of cell cycle progression resulted from insufficient function of the SKA complex during mitosis.

Down-regulation of DSN1 also increased G2/M arrest, but the duration of arrest was shorter than that observed after SKA3 knockdown (Figure 6C and 6D). DSN1 depletion did not increase the sub-G1 population, which was consistent with the result of apoptosis assay.

We then examined the expression of cyclins in SKA3- or DSN1-knockdown CRC cells (Supplementary Figure S8). Cyclin A and/or B1 expression were increased in both cell lines after SKA3 or DSN1 depletion (Supplementary Figure S8A-B); this was consistent with the increase in the G2/M phase cell population. Taken together, these results suggest that depletion of SKA3 or DSN1 affects the expression of cyclins, which in turn disturbs cell cycle progression.

### Depletion of SKA3 or DSN1 inhibited cell migration, invasion, and anchorage-independent cell growth in CRC cells

**Figure 7:**
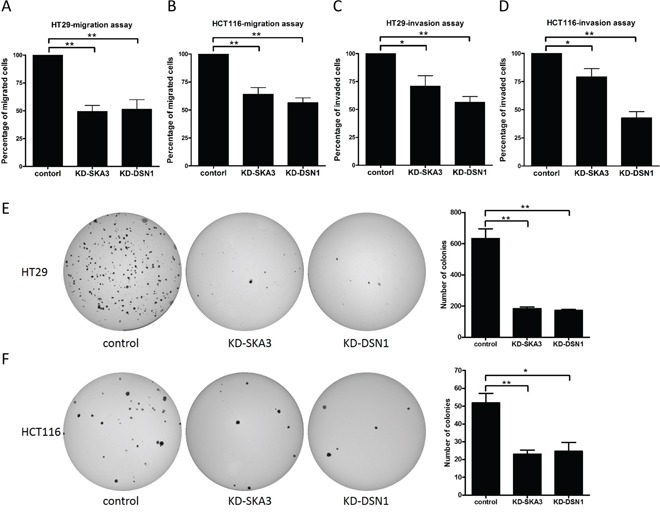
Knockdown of SKA3 or DSN1 inhibits migration, invasion, and anchorage-independent growth Transwell migration, invasion, and soft agar colony assays were conducted with HT29 and HCT116 cells transfected with control siRNA or target siRNA. Knockdown of SKA3 or DSN1 inhibited cell migration and invasion in both cell lines **A-D.** Relative percentages of migrated and invaded cells are presented as mean ± SD. Depletion of SKA3 or DSN1 decreased colony formation in **E.** HT29 cells and **F.** HCT116 cells. Representative images of colony formation following each treatment are shown on the left and quantification results are shown on the right. Numbers of colonies are presented as mean ± SD. (*p<0.05; **p<0.01).

To evaluate the roles of SKA3 and DSN1 in malignant transformation, we investigated the effects of knockdown on migration, invasion, and anchorage-independent cell growth (AIG) in HT29 and HCT116 cells. SKA3 or DSN1 knockdown decreased migration and invasion abilities in both cell lines (Figure 7A–7D). Interestingly, the decrease in invasiveness was stronger in DSN1-depleted cells than in SKA3-depleted cells (Figure 7C and 7D). Furthermore, in both cell lines, the number of colonies on soft agar was reduced when SKA3 or DSN1 was depleted (Figure 7E and 7F); this effect was also stronger in HT29 than in HCT116 cells.

## DISCUSSION

In this study, genomic and transcriptomic differences between CRC and CRA were identified using autologous tri-part samples. Although there is no guarantee that CRA samples were in fact precancerous CRC tissues, this study still provides more insight into CRC progression within individual patients than any previous studies. Through integrative analysis of chromosomal aberrations and differential gene expression, we identified 68 candidate genes that may be involved in malignant transformation. Among these, Aurora A, SKA3, and DSN1 protein levels were progressively upregulated in the tri-part samples. Intriguingly, there were no apparent associations between overexpression of the three proteins and clinicopathological carcinoma features. These results suggest that, although the up-regulation of these three proteins is critical for malignant transformation of adenoma into carcinoma, their levels may not change further during carcinoma progression.

Aurora A is overexpressed in colorectal cancer and is tightly associated with chromosome 20q gain [18, 30–32]. Sillars-Hardebol *et. al.* reported that Aurora A overexpression promoted chromosome 20q amplicon-driven progression of adenoma to carcinoma [18]. Our observation that Aurora A was progressively up-regulated during the progression of adenoma to carcinoma strongly suggests that Aurora A overexpression promotes malignant transformation during CRA development. Interestingly, Aurora A was up-regulated (fold change ≥ 1.5) in 54.9% (28 out of 51) of polyp samples, and 50% of these samples had a greater than 5-fold increase (Figure 2B). Because high Aurora A levels increase the speed of spindle fiber formation, which could lead to chromosomal instability in CRC cells [33], we speculate that the overexpression of Aurora A in polyps might increase CIN, in turn increasing susceptibility to malignant transformation. A growing number of Aurora A inhibitors have been developed, and some of them are being used in clinical trials [34]. Aurora A inhibitors may therefore serve as chemoprevention agents for patients with CRAs characterized by high Aurora A levels.

Amplification of chromosome 13q is also frequently observed in colon cancer [16, 30, 35]. However, the specific genes on this chromosome that contribute to malignant transformation are largely unknown. In this report, we identified *SKA3* at 13q12.11 as a novel gene that might be involved in malignant transformation of CRA. Pesson *et al.* have reported that *SKA3* mRNA is up-regulated in CRA and CRC tissues collected from different individuals [21]. Here, we further demonstrated that SKA3 protein was progressively up-regulated during progression from adenoma to carcinoma within individual patients. SKA3 is a subunit located in the kinetochore outer layer of the SKA complex, which works together with the NDC80 complex to control and promote proper mitotic exit during mitosis [36, 37]. Depletion of SKA3 induces mitotic arrest in HeLa cells [28, 29]. However, the oncogenic ability of SKA3 has not been studied in CRC cells. Here, SKA3 depletion in HT29 and HCT116 cells substantially reduced cell growth, increased apoptosis and cell cycle arrest, decreased migration and invasion ability, and decreased AIG. These effects were stronger in HT29 than in HCT116 cells, likely because hyperploidy in HT29 cells renders them more sensitive to reductions in SKA3 protein levels than diploid cells. Despite the differences in effect size, these results strongly suggest that SKA3 overexpression was important for overriding mitotic checkpoints in both CIN-high and CIN-stable cancer cells. Depletion of SKA1 or SKA2 in HT29 cells resulted in similar effects, suggesting that the SKA complex as a whole promotes malignant transformation.

Many anti-tumor reagents contribute to cell cycle arrest and subsequently lead to cell death [38–42]. Furthermore, synergistic effects have also been reported between agents targeting cell cycle modulators and chemotherapy drugs. Here, depletion of SKA1-3 increased not only G2/M arrest but also apoptosis in HT29 cells. These results suggest that drugs targeting the SKA complex may be useful treatments either alone or as adjuvants for sensitizing colorectal tumor cells to chemotherapeutic treatment. Additionally, inactivation of the SKA3 complex might be achieved by suppressing individual subunits or by disrupting interactions among the three subunits. These hypotheses are worthy of investigation in future *in vitro* and *in vivo* studies.

We also identified *DSN1*, the overexpression of which was driven by chromosome 20q gain, as an additional gene critically involved in tumor progression. Elevated DSN1 expression was positively associated with chr20 amplification, high CIN index, and SKA3 overexpression. DSN1 is required for proper kinetochore assembly and cell cycle progression. Defects in chromosome alignment and segregation were observed in HeLa cells with DSN1 depletion [43]. Interestingly, DSN1 knockdown resulted in a mild increase in G2/M arrest without affecting cell growth rates or apoptosis in both CRC cell lines. These results suggest that these cells can survive the mitotic defect caused by depletion of DSN1; the mechanism underlying this survival requires further investigation. By contrast, depletion of DSN1 significantly reduced malignant transformation in HT29 and HCT116 cells, as evidenced by reductions in migration, invasion, and AIG ability. Therefore, DSN1 may play other roles in CRC tumorigenesis in addition to maintaining proper kinetochore assembly.

In conclusion, this report provides clear evidence that overexpression of the *AURKA*, *SKA3*, and *DSN1* genes strongly correlates with the progression of CRA to CRC. Overexpression of these genes may lead to higher CIN in tumors and render them prone to malignant transformation. Therefore, patients who have polyps with high levels of Aurora A, SKA3, and/or DSN1 may require more thorough monitoring after polyp removal, as any residual cells may become malignant. Further investigation is required to identify the mechanism by which these kinetochore genes and their partner genes are initially up-regulated and how their overexpression leads to CIN in the specific chromosomes involved.

## MATERIALS AND METHODS

### Patient recruitment

Between 2010 and 2014, one hundred and fourteen CRC patients who underwent surgical resection for primary CRC were recruited in three collaborating hospitals: Kaohsiung Medical University Hospital, Tri-Service General Hospital, and Taipei Tzu Chi General Hospital. None of the patients were diagnosed with familial adenomatous polyposis (FAP), hereditary non-polyposis colorectal cancer (HNPCC), hamartomatous polyposis syndrome, Crohn's disease, or ulcerative colitis. In addition, none of them received chemotherapy or radiation prior to surgery. Fresh-frozen tissues, including carcinoma, paired adenomatous polyp, and paired non-neoplastic colon tissue located at least 10 cm away from the tumor lesions, were collected from all patients. The three autologous tissues are referred as “tri-part samples” throughout this report. Clinicopathological information for cancerous tissues and polyp tissues was collected whenever the information was available. We focused on the cases in which cancerous tissues were confirmed by pathological exam to be adenocarcinoma. A total of 76 patients were selected for screening analysis, and a summary of characteristics of this cohort is presented in Supplementary Table S1. A second cohort of 30 patients with known MSI statuses was included for immunoblotting analysis to increase statistical power. Histological sections of carcinoma and polyp samples were evaluated by experienced pathologists at each hospital. The study was approved by the Internal Review Boards at Academia Sinica and collaborating hospitals. Informed consent was obtained from all participants before information and samples were collected.

### DNA extraction, microsatellite instability assay, and chromosomal aberration detection

DNA was isolated from frozen tissues using a Gentra Puregene Tissue kit (Qiagen, Valencia, CA, USA) according to the manufacturer's instructions. The genomic DNA of tri-part samples was subjected to a microsatellite instability assay and chromosomal aberration detection. Details of the assays and data analysis are described in the Supplementary Materials and Methods. Briefly, 50 ng of genomic DNA was used for each PCR reaction that amplified from 5 microsatellite loci (BAT25, BAT26, D2S123, D5S346, and D17S250). The consensus guideline established by the National Cancer Institute for determining MSI status was used to determine the microsatellite instability of each tumor tissue. A total of 500 ng of genomic DNA from each sample was subjected to SNP genotyping using Genome-wide Human Array SNP6.0 (Affymetrix, CA, USA) according to the manufacturer's instructions. Genotyping was performed by the National Center for Genome Medicine at Academia Sinica, Taipei, Taiwan (http://ncgm.sinica.edu.tw). Copy number estimation was performed using Partek Genomics Suite (Partek Inc. MO, USA). Regions with copy number alteration (CNA) and the severity of chromosome instability (CIN) were determined for each sample as described in the Supplementary Materials and Methods.

### RNA isolation, RNA sequencing, data processing, and statistical analysis

Total RNA was extracted from frozen tissues using TRIzol (Life Technologies, Grand Island, NY, USA) following the manufacturer's instructions. The details of RNA sequencing, data processing, and statistical analysis are included in the Supplementary Materials and Methods. Briefly, RNA-seq was performed using a Ribo-Zero Magnetic Gold Kit (Human/Mouse/Rat) (Epicentre, Madison, WI, USA) and a TruSeq RNA Sample Preparation Kit (Illumina, San Diego, CA, USA) for library preparation and run on a HiSeq 2000 (Illumina, San Diego, CA, USA). Data cleaning, sequence alignment, and gene expression quantification were done sequentially using the corresponding software. Statistical analysis was performed for normalized expression levels using K-mean clustering and Wilcoxon signed-rank test.

### Cell culture

HCT116 cells were obtained from the Bioresource Collection and Research Center (BCRC, Taiwan). HT29 cells were a kind gift from Dr. Hsiu-Ming Shih (IBMS, Academia Sinica, Taiwan). HCT116 cells were maintained in McCoy's 5a medium and HT29 cells were cultured in DMEM. Both types of culture media were supplemented with 10% fetal bovine serum, 100 units/mL penicillin, 100 μg/mL streptomycin, and 1.5 mM L-glutamine.

### Protein extraction and immunoblotting

Frozen patient tissues were homogenized in 1X RIPA buffer (Millipore, Billerica, MA, USA) supplemented with 1X Complete protease inhibitor cocktails (Roche, Mannheim, Germany). The homogenates were briefly sonicated and centrifuged at 14000rpm at 4°C for 15 minutes. The supernatant (tissue lysate) was transferred to a new vial, and 30 μg of tissue lysate from each sample was resolved by 10% SDS-PAGE electrophoresis and then transferred to a PVDF membrane (Millipore, Billerica, MA, USA). The membrane was blocked with 5% skim milk in 1X TBST and then probed with primary antibodies at dilutions suggested by the manufacturers. Anti-DSN1, anti-SKA3, anti-UBE2C, anti-cyclin D, and anti-cyclin B1 antibodies were purchased from Abcam (Cambridge, MA, USA), anti-Aurora A was from Cell Signaling Technology (Danvers, MA, USA), anti-cyclin A and anti-cyclin E were from Santa Cruz (Santa Cruz, CA, USA), and anti-GAPDH was from Proteintech (Chicago, IL, USA). Immunoblotting images were quantified using GeneTools software (SynGene Inc., Frederick, MD, USA), and intensities of the target proteins were normalized to GAPDH.

### Immunohistochemistry (IHC) staining

IHC staining was performed on formalin-fixed paraffin-embedded tissue sections of the tri-part samples. The sections were deparaffinized, rehydrated, and endogenous peroxidase activity was blocked by incubating with 3% H_2_O_2_ for 10 minutes. Antigen retrieval was performed using pressure cooker heating in Citrate Buffer (Thermo Fisher Scientific, Fremont, CA, USA) for 10 minutes. The processed sections were incubated with primary antibody overnight at 4°C at the following dilutions: Aurora A: 1:250, SKA3: 1:800, DSN1: 1:3000 (Abcam, Cambridge, MA, USA). Immunostaining signals were enhanced and visualized using the ABC staining system and DAB substrate kit (Vector Laboratories, CA, USA) according to manufacturer's instructions. The slides were dehydrated and counterstained with hematoxylin. Signal intensity was scored as follows: 0 (no staining), 1 (weak staining), 2 (moderate staining), and 3 (strong staining). “Progressive increases” in protein levels were defined as carcinoma tissues having staining intensities higher than or equal to adenoma tissues, and both carcinoma and adenoma tissues having higher intensities than normal tissues.

### Transfection of siRNA

Transfection of the ON-TARGETplus siRNA pool (Dharmacon Research Inc., Lafayette, CO, USA) was carried out using Lipofectamine 2000 reagent (Life Technologies, Grand Island, NY, USA) according to the manufacturer's protocol. The final concentration of siRNA for each transfection reaction was 60 nM. ON-TARGETplus Non-targeting Control was used as a negative control. For each functional assay used to determine the biological effect of target gene knockdown, transfection reactions were performed in triplicate. The sequences of each siRNA are listed in Supplementary Table S8.

### Cell cycle analysis

Cells were plated in 6-well cell culture plates (3×10^5^ cells per well) and subjected to siRNA transfection. For cell cycle analysis, cells were collected two days after transfection by trypsinization, washed once with PBS, and incubated with 0.5 mL of Solution 10 containing 10 μg/mL 4′, 6-diamidino-2-phenylindole (DAPI) (ChemoMetec, Allerod, Denmark) at 37°C. After 5 minutes of incubation, 0.5 mL of solution 11 (ChemoMetec, Allerod, Denmark) was added and 30 μL of suspended cells were used for cell cycle analysis with a NucleoCounter NC-3000 (ChemoMetec, Allerod, Denmark).

### Cell growth curves

The growth rate of cells with siRNA-induced gene knockdown was measured using the iCelligence system (ACEA Bioscience, CA, USA). Briefly, one day after transfection, cells were seeded in 8-well cell culture plates (1.5×10^4^ cells per well) and cellular impedance was measured every 2 hours for 96 hours. Cell index was an arbitrary measurement derived from electrical impedance that reflected the number of living cells. All experiments were repeated independently three times.

### Transwell migration and invasion assays

The transwell migration assay was carried out using 24-well MILLIcell Hanging Cell Culture Inserts (8 μm) (Millipore, Billerica, MA, USA). Cells were transfected with target siRNA or control siRNA. One day after transfection, cells were harvested in serum free medium and seeded onto the upper compartment of the insert at a density of 5×10^4^ HCT116 cells or 2×10^5^ HT29 cells per insert. Complete medium containing 10% FBS was used as a chemoattractant in the lower compartment. After one day of incubation at 37°C, the membrane surface was fixed with methanol and the cells on the upper compartment were removed using cotton swabs. The migrated cells were visualized by Giemsa staining and counted under a microscope. For the invasion assay, harvested cells were seeded on HTS FluoroBlok transwells coated with extracellular matrix gel (Corning, Bedford, MA, USA); complete medium was added to the lower compartment as an attractant. After 24 hours of incubation at 37°C, the invaded cells were visualized by staining with Hoechest 33342 and quantified under a fluorescent microscope. Data were collected from three independent experiments.

### Soft agar colony formation assay

Cells were transfected with target siRNA or control siRNA. One day after transfection, 6000 cells were re-suspended in DMEM supplemented with 10% FBS and 0.25% top agarose and plated onto wells pre-layered with 0.7% bottom agarose mixed with DMEM/10% FBS in 6-well cell culture plates. The plates were incubated for 4 weeks at 37°C and the medium was replaced every 3 days. Colonies were visualized by staining with 0.1% crystal violet (Sigma-Aldirich, St Louis, MO, USA) and counted manually. All experiments were repeated independently three times.

### Statistical analysis

Hierarchical clustering of samples and differentially expressed genes identified by RNA-seq was performed with Euclidean distance and average-linkage as a measurement of dissimilarity. Pearson correlations were used to determine the correlation between mRNA expression levels detected by deep sequencing (expressed as fold change) and protein expression levels by immunoblotting (expressed as fold change). Paired sample *t*-tests were used to determine differences in the relative expression levels of a protein between carcinoma samples and paired polyps. If the normalized fold change in protein expression in either the polyp or carcinoma sample of a patient was more than 3 standard deviations above the mean for all MSS tumors, the patient was regarded as an outlier and excluded from statistical analysis. Wilcoxon rank-sum test was performed to test associations between protein expression levels and clinicopathological features. Fisher's exact test was performed to test the association between protein expression levels and molecular features of tumor samples. Two-sample *t*-tests were performed to test the significance of the biological effects of gene knockdown. Analyses were carried out using SAS version 9.3 (SAS Institute, Cary, NC, USA). Two-tailed *p*-values < 0.05 were considered statistically significant.

## SUPPLEMENTARY DATA, FIGURES AND TABLES, REFERENCES




