# Supplementary material for: Over-expression of AURKA, SKA3 and DSN1 contributes to colorectal adenoma to carcinoma progression
Source: Oncotarget. 2016 Jun 13;7(29):45803–18. doi: 10.18632/oncotarget.9960 (PMC5216762; doi:10.18632/oncotarget.9960)
Supplement: Supplementary file 1 [file oncotarget-07-45803-s001.pdf]

Over-expression of *AURKA*, *SKA3* and *DSN1* contributes to colorectal adenoma to carcinoma progression

SUPPLEMENTARY DATA

Microsatellite instability assay

Fifty ng of genomic DNA from 106 carcinomas, 99 polyps, and 106 paired non-neoplastic colon tissues was used for each PCR reaction. PCR products amplified from 5 microsatellite loci (BAT25, BAT26, D2S123, D5S346, and D17S250) were analyzed by capillary electrophoresis using an ABI 3730 DNA analyzer (Life Technologies, Grand Island, NY). The consensus guideline established by the National Cancer Institute for determining MSI status was used to determine microsatellite instability of each tumor tissue. DNA samples with microsatellite instability were categorized as follows: MSI-high (MSI-H), instability at ≥2 loci; MSI-low (MSI-L), instability at one locus; microsatellite stable (MSS), no detectable instability.

Chromosomal aberration detection

A total of 500 ng of genomic DNA each from 76 carcinoma, 67 polyp, and 76 paired non-neoplastic colon tissue samples was subjected to SNP genotyping using Genome-wide Human Array SNP6.0 (Affymetrix, CA, USA) according to the manufacturer's instructions. Genotyping was performed by the National Genotyping Center at Academia Sinica, Taipei, Taiwan (http://ngc.sinica.edu.tw). Copy number estimation for carcinoma tissue and polyp tissue was performed using Partek Genomics Suite (Partek Inc. MO, USA) under paired mode by comparing probe intensity data from neoplasia tissues to that from corresponding normal colon tissues to filter out germ-line copy number variations (CNV). Regions with at least 50 consecutive probes with inferred copy numbers > 2.2 or < 1.8 were defined as having a copy number alteration (CNA). A given chromosome arm with CNAs detected in more than 50% of the total length was defined as having amplification or deletion of the whole chromosome arm. In this report, the severity of chromosome instability (CIN) was classified into five categories: CIN-stable (no altered chromosome arms), low degree of CIN (≥ 1 but ≤ 5 altered chromosome arms), medium degree of CIN (> 5 but ≤ 10 altered chromosome arms), high degree of CIN (> 10 but ≤ 20 altered chromosome arms), and ultra-high degree of CIN (> 20 atlered chromosome arms).

RNA sequencing and data processing

Tri-part samples from 10 patients were subjected to massively parallel sequencing for expression profiling and quantification. RNA quality and quantity was checked using an Agilent Bioanalyzer. One µg of total RNA from each sample was treated with the Ribo-Zero Magnetic Gold Kit (Human/Mouse/Rat) (Epicentre, Madison, WI, USA) according to the manufacturer's instructions to remove ribosomal RNA. The purified RNA was subjected to sequencing library construction using the TruSeq RNA Sample Preparation Kit (Illumina, San Diego, CA, USA) with the mRNA selection step skipped. RNA libraries were sequenced on a Hiseq 2000 instrument (Illumina, San Diego, CA, USA) using Illumina's TruSeq PE Cluster kit v3 and TruSeq SBS kit v3 for paired-end 100bp sequencing. Libraries were spiked with approximately 1% of the PhiX control library according to Illumina's recommendation.

Quality control of the raw sequence data was performed in two steps. First, adapters and low quality reads with Phred quality scores (Q score) < 13 were trimmed. Then, processed reads with lengths < 25 bp were removed by Solexa QA (version 2.5) [1]. The qualified paired-end reads were mapped to Human reference genome hg19 using Bowtie (version 1.0.1) in parallel with Tophat (version 2.0.11) to analyze the mapping results for splice junction identification between exons [2, 3]. The Sequence Alignment/Map (SAM) file for each sample was quantified and normalized to estimate gene expression levels using Cufflinks (version 2.2.1) with default options [4]. Gene expression levels in fragments per kilobase of transcript per million mapped reads (FPKM values) were used for further statistical analysis.

Statistical analysis of RNA-seq data

Since the polyp sample from patient CRC10 did not pass FASTQC, tri-part samples from this patient were excluded from downstream analysis. A total of 23,615 genes were reported by Cuffmerge and were further filtered by expression abundance. Only genes with detectable expression in more than 50% of samples and with average FPKM ≥ 1 were included in statistical analysis. A total of 14,516 genes passed this filter. The Multi-Omics On Line

Analysis System (MOLAS) (http://molas.iis.sinica.edu.tw/) was used to perform K-mean clustering analysis for the 14,516 genes with the following parameters: p-value ≤ 0.001, fold change ≥ 2, clustering # = 9, min_row_sum # = 1, and dispersion # = 0.001. Genes in the clusters showing progressive expression level increases or decreases from non-neoplastic colon tissue through polyps to carcinomas, and with mean FPKM ≤ 1 for non-neoplastic tissue or mean FPKM ≥ 1 for carcinoma, were considered candidate genes. The Wilcoxon Signed Rank test was performed for the same 14,516 genes in a pair-wise manner using SAS version 9.3 (SAS Institute, Cary, NC, USA). Genes with p-values ≤ 0.01 and fold change > 2 or < -2 in carcinoma compared to paired non-neoplastic tissue, and with p-values ≤ 0.01 and fold change > 1.5 or < -1.5 in carcinoma compared to paired polyp, were considered differentially expressed. Pathway enrichment analysis of the candidate genes was performed using Ingenuity Pathway Analysis software (Qiagen, Valencia, CA, US)

Genomic real-time quantitative PCR

Real-time quantitative PCR was performed using Power SYBR Green master mix (Applied Biosystems, Foster City, CA, USA). Briefly, PCR reactions with 2 ng of genomic DNA template were run on an ABI PRISM7900 using the default 2 step thermal cycle comprised of a heat activation step at 95ºC for 10 min, 40 cycles of 95ºC for 15 s and 60ºC for 1 min, and a dissociation stage at the end. The *NSE1* gene located on chromosome 2, which was the least frequently altered among all tissue samples, served as an internal control. Two primer pairs specific to exon 4 and exon 6 of *SKA3* were used to evaluate *SKA3* copy number. The primer sequences were as follows:

NSE1 forward, 5'-GTGTCTGGGAACTTGAATGTG-3',

NSE1 reverse, 5'-TGCTGGTTTCTGGAATGGTG-3';

SKA3-1 forward, 5'-ATGATCTGTCTGATCCTCCTGTTG-3',

SKA3-1 reverse, 5'-GGATACGATGTACCGCTCAAGTC-3';

SKA3-2 forward, 5'-TTTTATGTGTCTTTTAGATGCCGAAT-3',

SKA3-2 reverse, 5'-AAGCTATGCTGTTCTTTGTAGATGGA-3'.

All reactions were carried out in triplicate. *SKA3* copy numbers for each tumor sample are expressed as $2x2^{-\Delta\Delta Ct}$ compared to the paired non-tumor sample. Copy numbers higher than 2.2 indicated gene amplification. The *SKA3* gene was considered amplified when amplification was detected by both primer pairs. Samples with suspected *NSE1* amplification detected by the SNP genotyping array were excluded from statistical analysis.

Apoptosis analysis

Cells were plated in 6-well cell culture plates ($3x10^5$ cells per well) and subjected to siRNA transfection. Attached and floating cells were harvested after 48 hours and washed once with PBS. Harvested cells were resuspended in Annexin V binding buffer containing Annexin V-Alexa Fluor 488 conjugate and Hoechest 33342 (Life Technologies, Grand Island, NY). After 15 minutes of incubation at 37ºC, cells were washed once with binding buffer and resuspended in Annexin V binding buffer containing PI. The stained cells were analyzed with a NucleoCounter NC-3000. Raw data were exported and analyzed using FlowJo software (Ashland, OR, USA). Data were collected from three independent experiments.

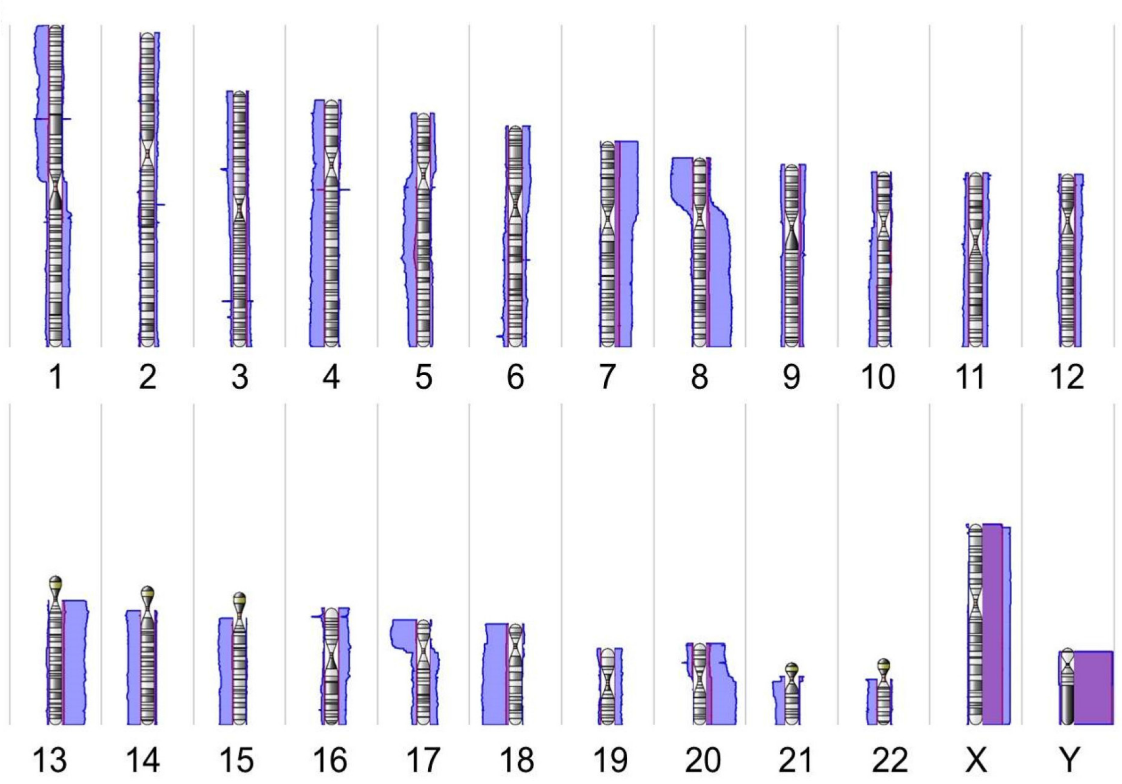

Supplementary Figure S1: Karyogram view of accumulated copy number alterations in polyp samples and carcinoma samples. Purple bars indicate CNAs detected in polyp samples and blue bars indicate CNAs detected in carcinoma samples. Bars on the right side represent amplification-type CNAs while bars on the left side represent deletion-types. The height of the bars indicates the accumulated samples with CNAs in that region. Chromosomes numbers are indicated at the bottom of each karyogram.

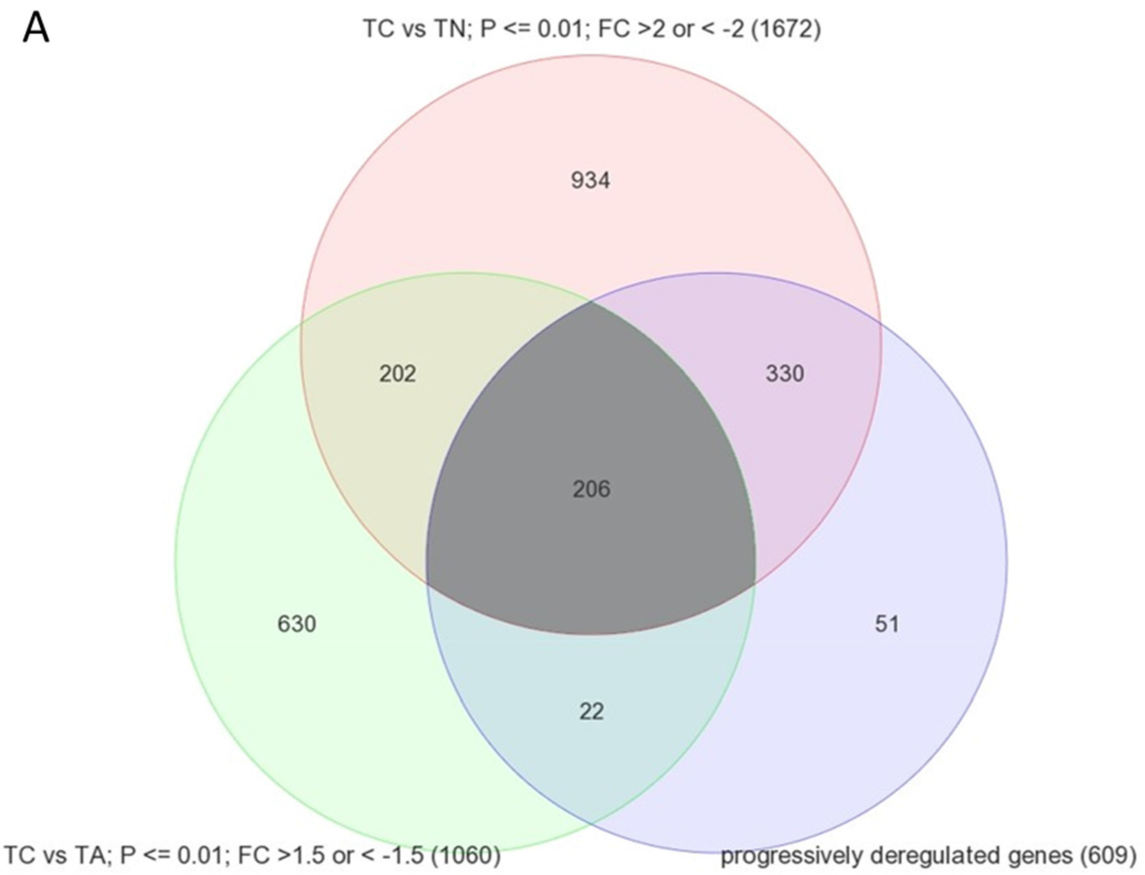

Supplementary Figure S2: Identification of malignant transformation-related genes. A. Venn diagram of the genes identified by Wilcoxon signed rank test and k-mean clustering. 1,060 (TC V.S. TA) and 1,672 (TC V.S. TN) differentially expressed genes passed the indicated filters. 609 progressively deregulated genes were identified by the k-mean clustering method. A total of 206 deregulated genes (located in the gray area in the center of Venn diagram) were included in all three gene lists. (FC, fold change; TC, carcinoma tissue; TA, polyp tissue; TN, paired non-neoplastic colon tissue).

(*continuued*)

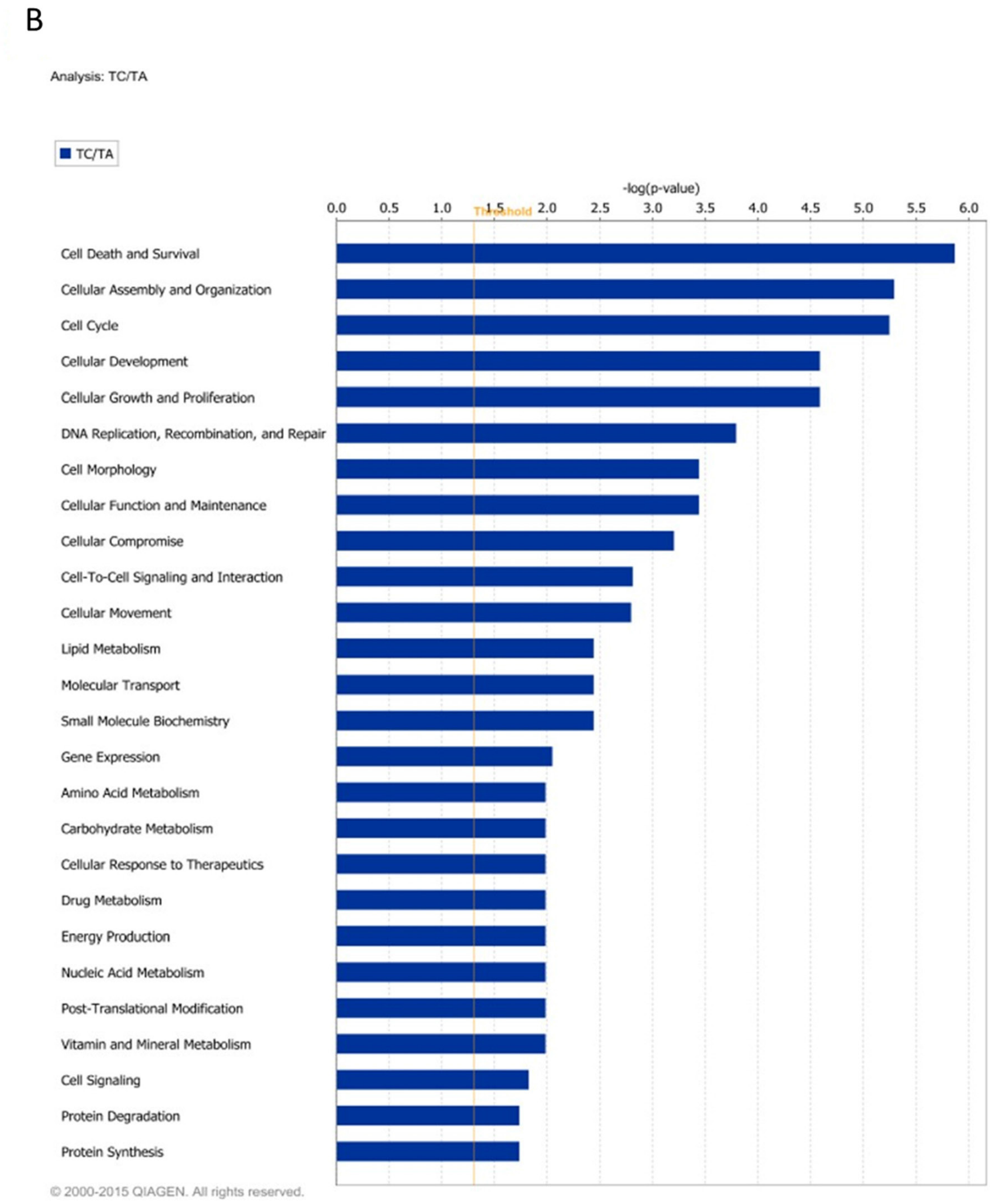

Supplementary Figure S2 (*continued*): B. Results of Ingenuity Pathway Analysis for the 206 candidate malignant transformation-related genes. 26 significant molecular and cellular functions with $p \leq 0.05$ are presented. The top 3 biological activities were cell death and survival, cellular assembly and organization, and cell cycle.

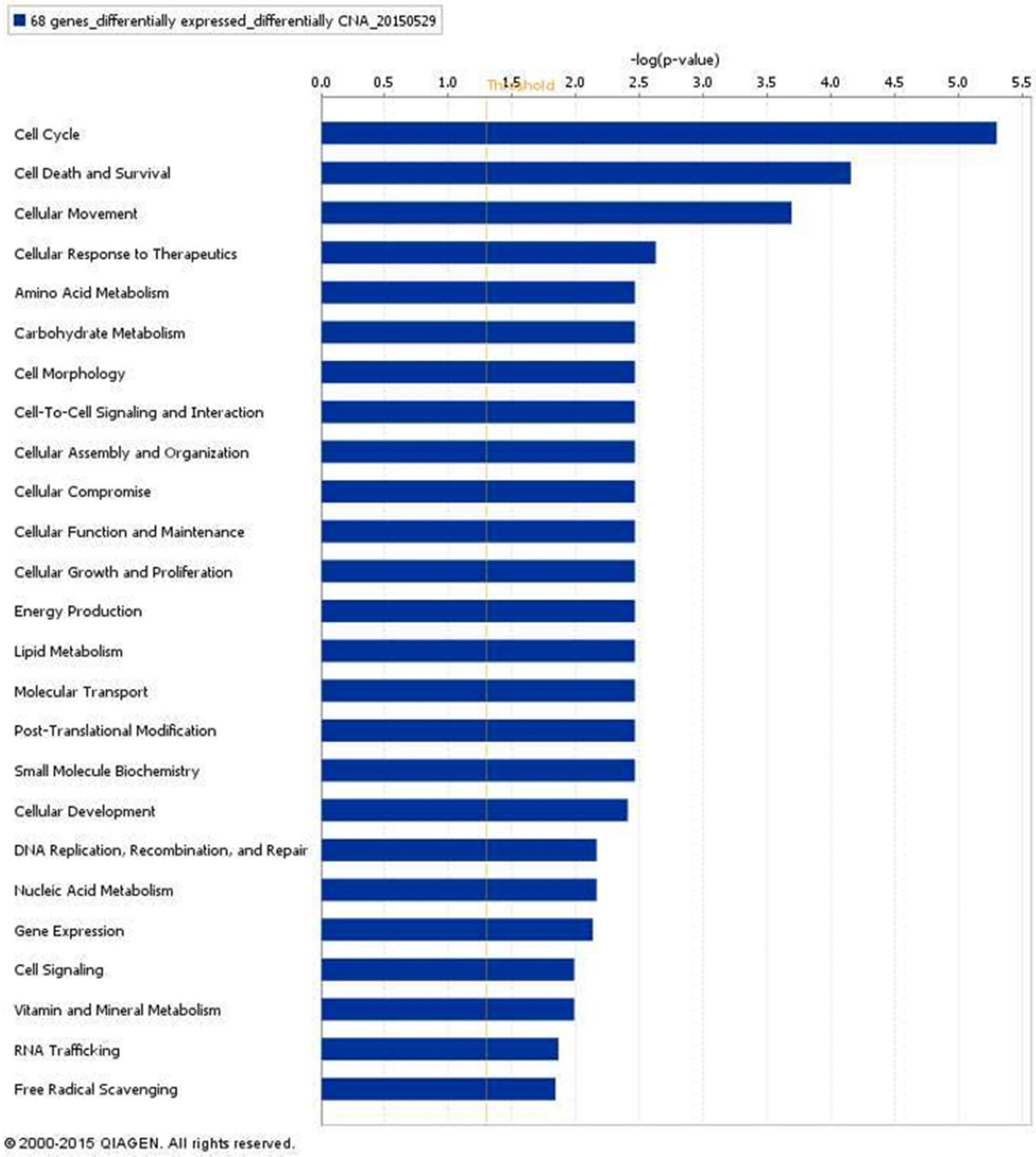

Supplementary Figure S3: Ingenuity Pathway Analysis of the 68 candidate genes. 25 significant molecular and cellular functions with $p \leq 0.05$ were identified. The top 3 biological activities were cell cycle, cell death and survival, and cellular movement.

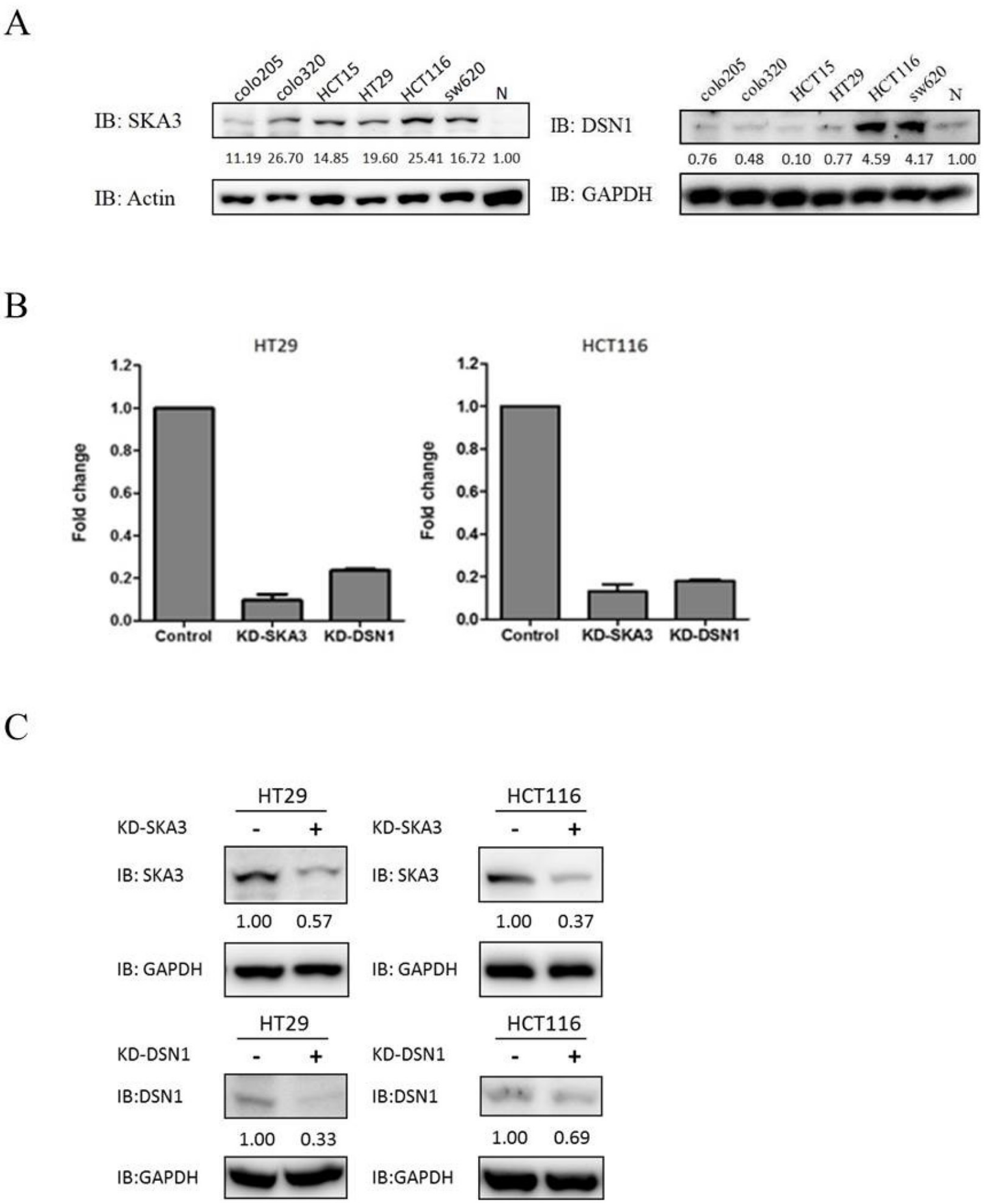

Supplementary Figure S4: Knockdown efficiency of siSKA3 and siDSN1. Efficiency of siRNA-mediated knockdown was determined by RT-qPCR using *SKA3* and *DSN1* specific primer pairs to evaluate mRNA levels and immunoblotting analysis to evaluate protein levels. **A.** Endogenous SKA3 and DSN1 protein expression in six CRC cell lines. Actin and GAPDH served as internal controls for protein loading. Relative fold changes of protein expression levels were determined by comparison to N and are shown under each lane (N, pooled non-neoplastic colon tissues from five patients). **B.** mRNA expression of *SKA3* and *DSN1* was reduced by more than 75% in HT29 and HCT116 cells transfected with indicated siRNA. **C.** Protein expression was decreased in siRNA-transfected HT29 and HCT116 cells. Representative images are shown. Relative fold changes in protein expression level are shown under each knockdown condition.

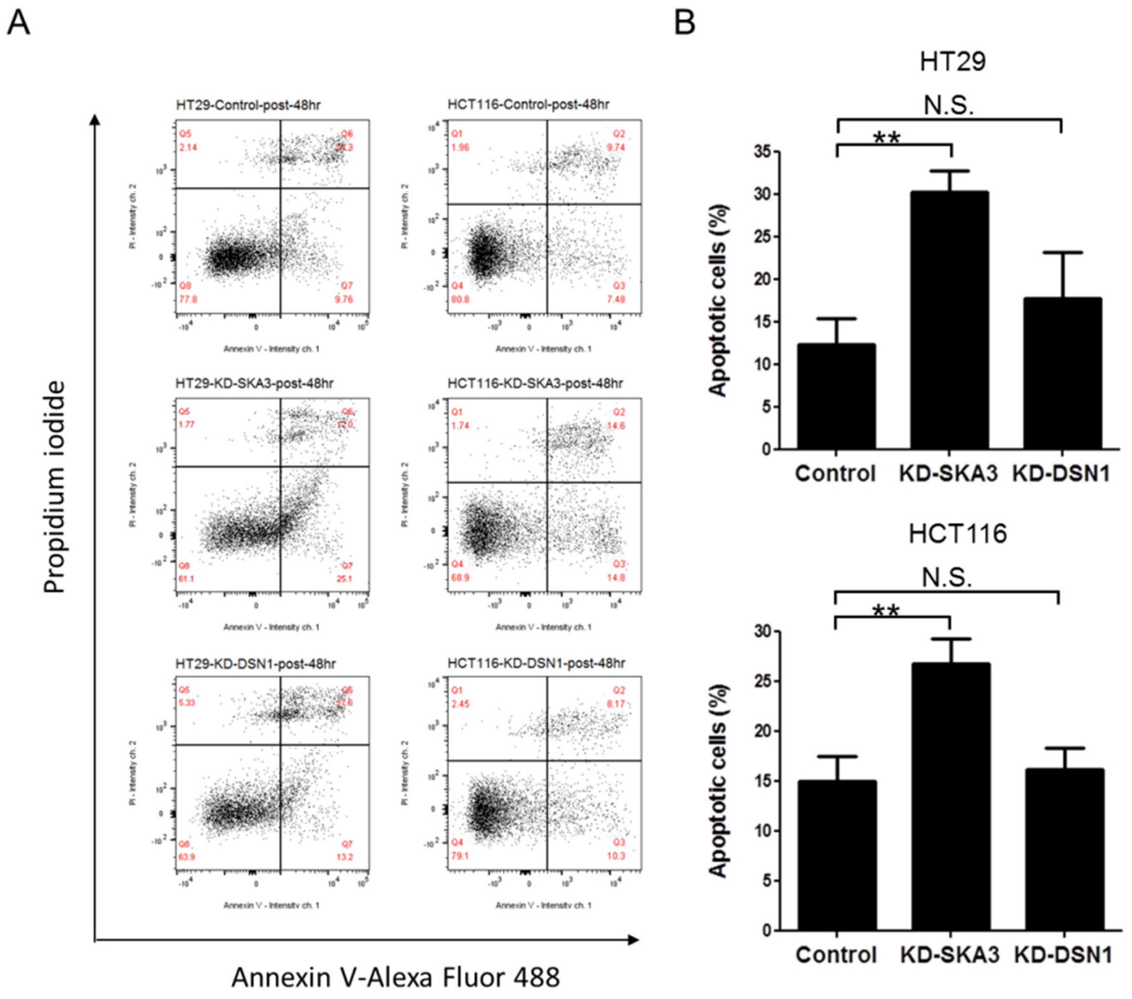

Supplementary Figure S5: Knockdown of SKA3 increases cell apoptosis. HT29 and HCT116 cells were transfected with control siRNA or target siRNA. Two days post-transfection, cells were collected for Annexin V-Alexa Fluor-488 conjugate/Propidium Iodide (PI) staining and analyzed using NucleoCounter. The percentage of apoptotic cells (Annexin V^+/PI^- + Annexin V^+/PI^+) for each condition is presented as mean ± SD. (**p<0.01; N.S., not significant) **A.** A typical result is shown, with control siRNA in the top panel and target siRNA in the middle and bottom panels. **B.** Averaged results of three independent experiments are presented. Knockdown of SKA3 in HT29 and HCT116 cells increased the apoptotic cell population. Knockdown of DSN1 tended to slightly increase apoptotic cell populations in HT29 cells, but this result did not reach statistical significance.

A

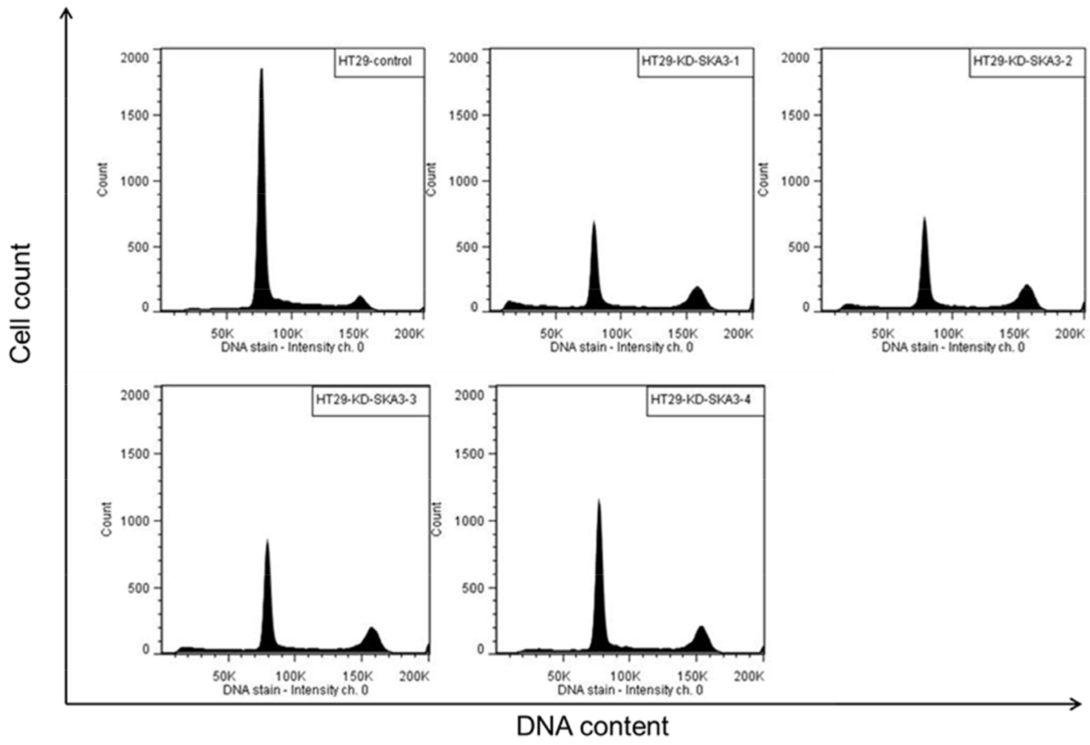

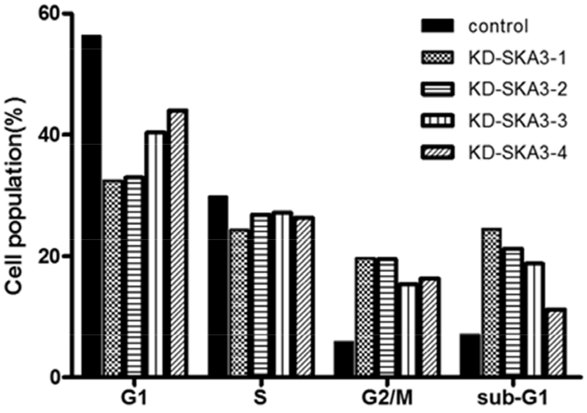

Supplementary Figure S6: Knockdown of SKA3 with 4 individual siRNA oligos affects cell cycle progression. A. Knockdown of SKA3 with individual anti-SKA3 siRNAs. siSKA3-1 (J-015700-5), siSKA3-2 (J-015700-6), siSKA3-3 (J-015700-7), or siSKA3-4 (J-015700-8) in HT29 cells decreased the G1 phase population and increased the sub-G1 and G2/M populations.

(*continuued*)

B

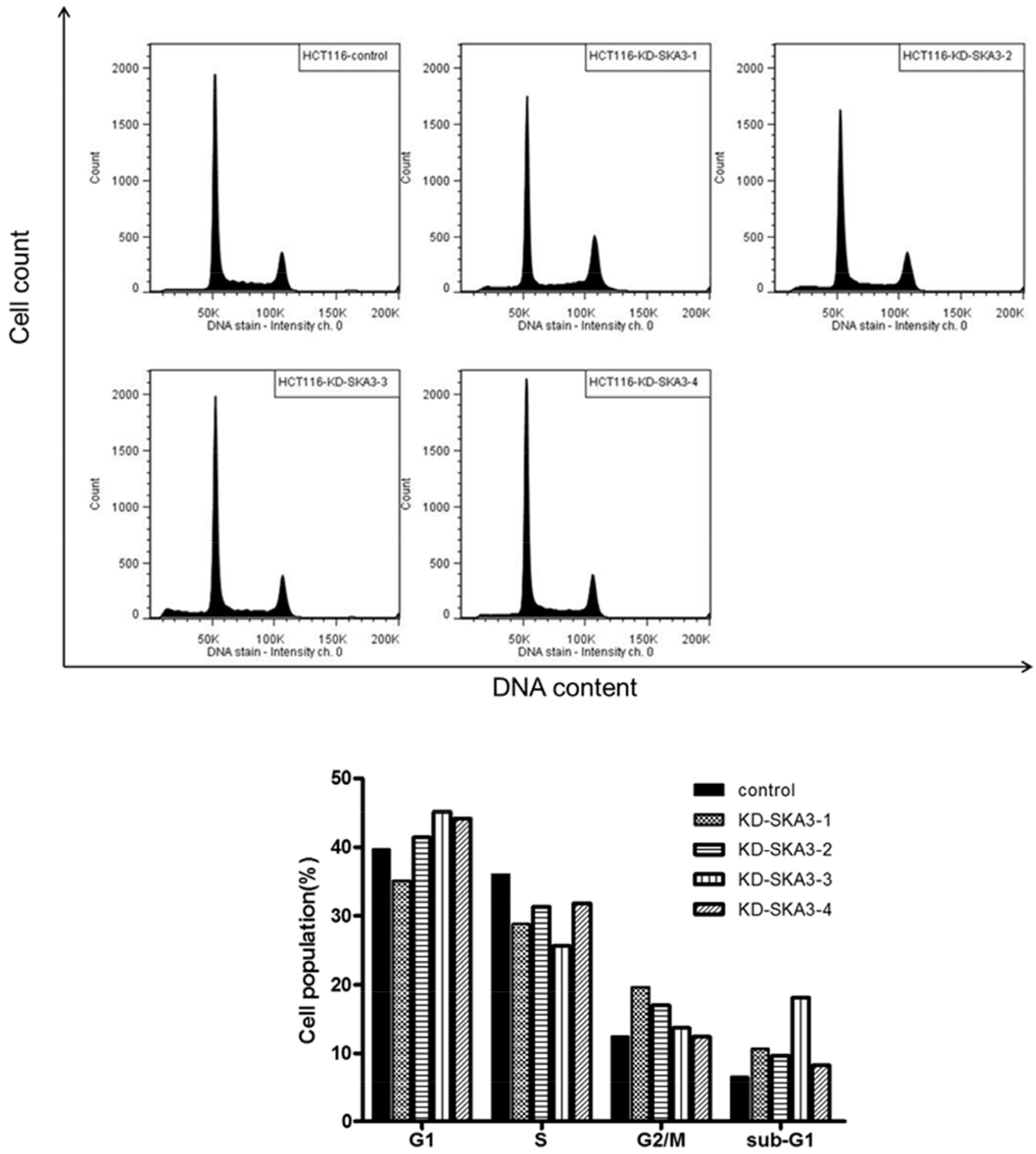

Supplementary Figure S6 (*continued*): B. Knockdown of SKA3 with siSKA3-1, siSKA3-2, siSKA3-3, or siSKA3-4 in HCT116 cells decreased the S phase population and increased the sub-G1 and G2/M populations.

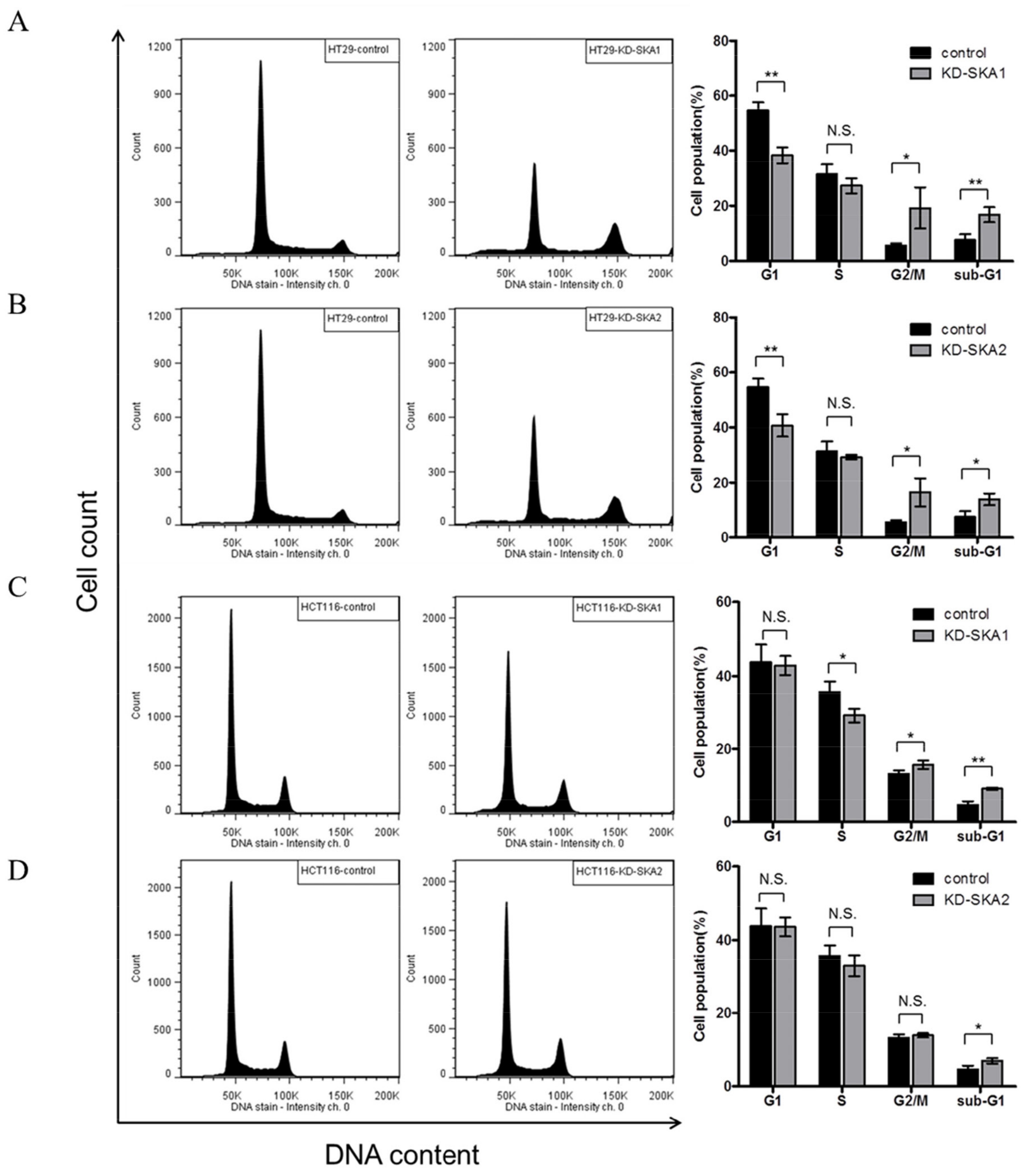

Supplementary Figure S7: Knockdown of SKA1 or SKA2 affects cell cycle progression. HT29 and HCT116 cells were transfected with control siRNA or target siRNA. 48 hours post-transfection, cells were collected for DAPI staining and cell cycle distribution analysis. A typical cell cycle distribution result is shown, with control siRNA in the left column and target siRNA in the middle column. Averaged results of three independent experiments are presented in the right column. Percentages of cells in each cell cycle phase are shown as mean ± SD. (*p<0.05; **p<0.01; N.S., not significant) **A, B.** Knockdown of SKA1 or SKA2 in HT29 cells decreased the G1 phase population and increased the G2/M and sub-G1 populations. **C.** Knockdown of SKA1 in HCT116 cells decreased the S phase population and increased the G2/M and sub-G1 populations. **D.** Knockdown of SKA2 in HCT116 cells increased the sub-G1 population.

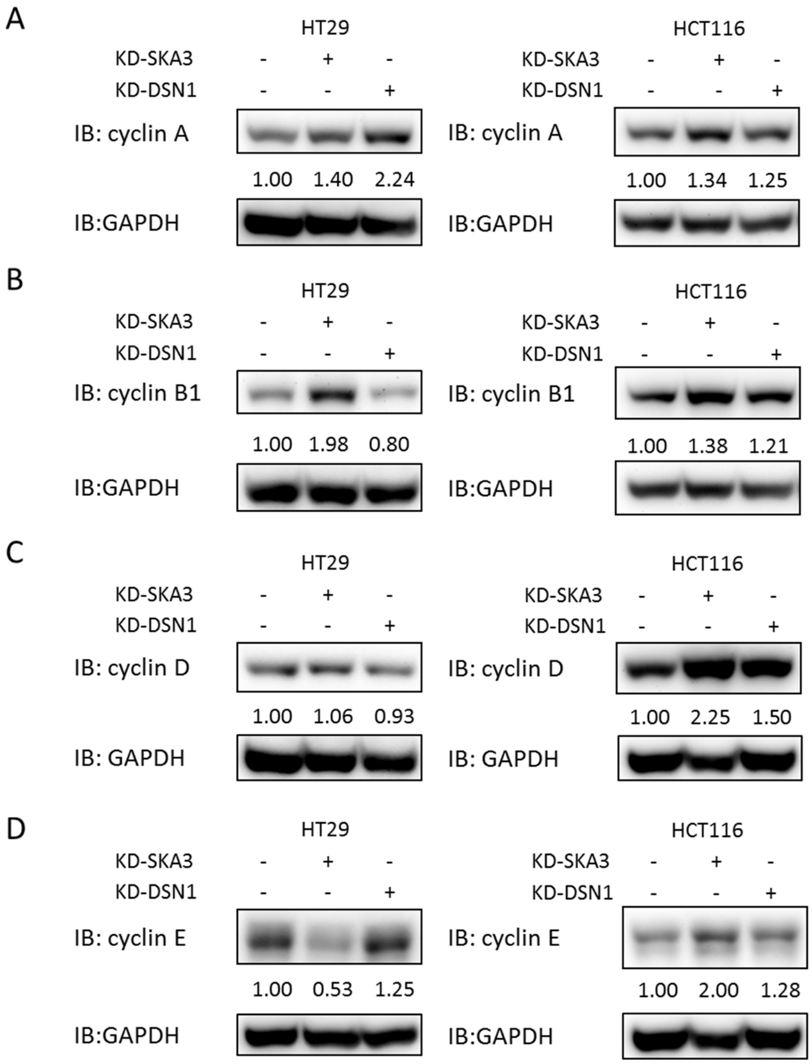

Supplementary Figure S8: Knockdown of SKA3 or DSN1 affects cyclin expression. Immunoblotting assay was done for HT29 and HCT116 cells transfected with control siRNA or target siRNA. **A.** Target gene knockdown increased cyclin A protein expression in both cell lines. **B.** SKA3 depletion increased cyclin B1 levels in both cell lines. DSN1 knockdown slightly increased cyclin B1 levels in HCT116 cells and slightly decreased cyclin B1 levels in HT29 cells. **C.** Knockdown of either SKA3 or DSN1 increased cyclin D levels in HCT116 cells. **D.** Depletion of SKA3 increased cyclin E levels in HCT116 cells, but reduced cyclin E levels in HT29 cells.

Supplementary Table S1: Characteristics of the screening cohort

	**Number of subjects (%)**
Total patients	76 (100.0%)
Gender	
Male	40 (52.6%)
Female	36 (47.4%)
Mean age, years (range)	69 (37-87)
Location of cancer	
Ascending colon	20 (26.3%)
Transverse colon	6 (7.9%)
Descending colon	4 (5.3%)
Sigmoid	28 (36.8%)
Rectum	18 (23.7%)
TNM stage	
Stage 0	1 (1.3%)
Stage I	13 (17.1%)
Stage II	20 (26.3%)
Stage III	34 (44.7%)
Stage IV	8 (10.5%)
MSI status	
MSS	63 (82.9%)
MSI-L	5 (6.6%)
MSI-H	8 (10.5%)
CIN status*	
Stable (0)	10 (13.2%)
Low degree (1)	10 (13.2%)
Medium degree (2)	9 (11.8%)
High degree (3)	30 (39.5%)
Ultra-high degree (4)	17 (22.4%)

*, Stable (0): 0 altered chromosome arms; Low degree (1): >1 but ≤5 altered chromosome arms; Medium degree (2): >5 but ≤10 altered chromosome arms; High degree (3): >10 but ≤20 altered chromosome arms; Ultra-high degree (4): >20 altered chromosome arms.

Supplementary Table S2: Significant diseases and bio-functions associated with the 206 identified genes

Diseases or Functions Annotation	*p-value*	Predicted Activation State	Activation z-score	# Molecules
solid tumor	1.72E-10		1.038	61
malignant solid tumor	1.49E-09		1.091	57
cohesion of sister chromatids	5.14E-06		1.192	4
cell survival	2.63E-05		1.227	28
cell death of breast cancer cell lines	3.04E-05		1.571	14
G2/M phase	4.22E-05		1.938	8
cell viability	7.59E-05		1.649	26
G2 phase	8.82E-05		1.482	9
amplification of centrosome	1.61E-04		1.067	4
cell viability of tumor cell lines	2.89E-04		1.669	21
G2 phase of tumor cell lines	3.56E-04		1.482	7
S phase	4.64E-04		1.091	8
cell death of melanoma cell lines	1.62E-03		1.19	9
interphase of tumor cell lines	2.52E-03		1.482	11
cell viability of prostate cancer cell lines	3.04E-03		1.067	4
G2/M phase of tumor cell lines	4.59E-03		1.938	4
transport of molecule	5.03E-03		1.367	8
accumulation of cells	5.39E-03		-1.067	4
apoptosis of melanoma cell lines	5.73E-03		1.482	5
cell movement of colon cancer cell lines	6.89E-03		1.982	5
interphase	7.04E-03		1.531	12
DNA replication	7.97E-03		1.718	4
cell movement of tumor cell lines	8.12E-03		1.318	18
ploidy	9.93E-03		1.992	4
autophagy of tumor cell lines	1.07E-02		-1.091	5
synthesis of DNA	1.11E-02		-1.158	7
metabolism of DNA	1.15E-02	Increased	2.193	6
cell viability of cervical cancer cell lines	1.18E-02		1.353	7
cell death of bone cancer cell lines	1.30E-02		1.109	6
differentiation of tumor cell lines	1.40E-02		1.082	7
apoptosis of ovarian cancer cell lines	1.82E-02		1.109	4
colony formation of tumor cell lines	2.67E-02		-1.172	7
proliferation of cancer cells	2.80E-02		1.342	5
cell death of immune cells	2.95E-02		-1.858	7

Disease or function pathways with P <= 0.05 and activation Z-score >= 1 or <= -1 are reported.

Supplementary Table S3: List of genes with differential copy number alterations and progressively deregulated expression

See Supplementary File 1

Supplementary Table S4: IHC scores of CRC patient tri-part samples

Aurora A					SKA3					DSN1				
**sample ID**	**N**	**A**	**C**	**progressive up-regulation**	**sample ID**	**N**	**A**	**C**	**progressive up-regulation**	**sample ID**	**N**	**A**	**C**	**progressive up-regulation**
CRC010	0	1	1	Y	CRC010	0	1	3	Y	CRC011	0	0	1	Y
CRC012	1	1	3	Y	CRC011	1	2	2	Y	CRC014	0	0	1	Y
CRC013*	0	0	2	Y	CRC012	1	0	2	N	CRC015*	0	0	1	Y
CRC014	0	2	2	Y	CRC014	0	1	1	Y	CRC018	0	0	1	Y
CRC015*	0	0	2	Y	CRC015*	0	0	1	Y	CRC021	0	0	1	Y
CRC016	0	1	1	Y	CRC018	1	1	2	Y	CRC023	0	0	1	Y
CRC017	0	2	2	Y	CRC022	1	1	3	Y	CRC028	0	1	1	Y
CRC019	1	1	3	Y	CRC023	0	1	3	Y					
CRC020	0	0	1	Y	CRC024	0	1	3	Y					
CRC022	0	0	3	Y	CRC025	1	0	1	N					
CRC023	0	2	3	Y	CRC027	0	0	1	Y					
CRC024	1	2	3	Y	CRC028	1	3	2	N					
CRC026	0	2	1	N	CRC029*	1	2	3	Y					
CRC027	0	1	1	Y										
CRC029*	0	2	3	Y										

N, non-neoplastic tissue; A, polyp; C, carcinoma. Signal intensity was scored as follows: 0 (no staining), 1 (weak staining), 2 (moderate staining) and 3 (strong staining). *, Hyperplastic polyps; all others were adenomatous polyps.

Supplementary Table S5: Analysis of associations between clinicopathological features and protein expression levels in adenoma samples

Pathological features	Aurora A		SKA3		DSN1	
	Number of samples[&]	*p*-value	Number of samples[&]	*p*-value	Number of samples[&]	*p*-value
Size of adenoma	34	0.4843	43	0.8346	43	0.3644
≥1cm	23		32		31	
<1cm	11		11		12	
Grade of dysplasia	33	0.7824	43	0.4943	43	0.5167
mild + moderate	28		37		37	
severe	5		6		6	

[&], Adenoma samples with MSI, adenocarcinoma nodules or extreme alteration of protein expression were excluded from analysis.

Supplementary Table S6: Analysis of associations between clinicopathological features and protein expression levels in carcinoma samples

Clinicopathological features	Aurora A		SKA3		DSN1	
	Number of samples[&]	*p-value*	Number of samples[&]	*p-value*	Number of samples[&]	*p-value*
Gender	52	0.8828	67	0.1561	71	0.5148
Male	29		30		38	
Female	23		37		33	
Age at diagnosis	52	0.2845	67	0.3308	71	0.9945
Age≤60	15		17		16	
Age>60	37		50		55	
Location of carcinoma	52	0.9918	67	0.1433	71	0.8813
Colon	38		49		52	
Rectum	14		18		19	
Size of carcinoma	52	0.9352	67	0.4926	71	0.1845
≥5cm	10		14		17	
<5cm	42		53		54	
cTNM stage[#]	51	0.1040	66	0.8017	70	0.5544
I+II	22		30		31	
III+IV	29		36		39	
Primary tumor (T)[*]	52	0.5454	67	0.9508	71	0.6188
T1 + T2	16		20		21	
T3 + T4	36		47		50	
Regional lymph nodes (N)[*]	52	0.0507	67	0.8352	71	0.6166
Yes	24		30		34	
No	28		37		37	

[&], Carcinoma samples with MSI or extreme alteration of protein expression were excluded from analysis. [#], cTNM is the clinical classification. *, Stage according to pathological examination (pTNM).

Supplementary Table S7: Analysis of associations between genomic and molecular alterations and protein overexpression status in polyp and carcinoma samples from the screening set

Genomic alterations	Aurora A				SKA3				DSN1			
	Fold change ≧ 1.5	Fold change < 1.5	Total[#]	*p-value*	Fold change ≧ 1.5	Fold change < 1.5	Total[#]	*p-value*	Fold change ≧ 1.5	Fold change < 1.5	Total[#]	*p-value*
CIN status*			89				103				99	
0,1	31	17	48	0.0056	30	26	56	0.0228	14	39	53	0.0010
2,3,4	37	4	41		36	11	47		28	18	46	
CIN in tumor			89				103				99	
Positive	46	10	56	0.1231	44	19	63	0.1442	31	29	60	0.0238
Negative	22	11	33		22	18	40		11	28	39	
Loss of heterozygosity			89				103				99	
Positive	23	44	67	0.4291	26	7	33	0.0469	19	23	42	0.0154
Negative	5	17	22		40	30	70		12	45	57	
Chr13 gain			89				103				99	
Yes	26	4	30	0.1211	22	10	32	0.6577	22	12	34	0.0015
No	42	17	59		44	27	71		20	45	65	
SKA3 amplification[&]			83				97				94	
Yes	24	4	28	0.1168	22	8	30	0.2546	16	14	30	0.1142
No	38	17	55		40	27	67		22	42	64	
Chr20 gain			89				103				99	
Yes	39	6	45	0.0259	35	14	49	0.1551	30	19	49	0.0002
No	29	15	44		31	23	54		12	38	50	
Protein overexpression												
Aurora A fold change ≥ 1.5							81				81	
Yes	---	---			48	18	66	0.0305	26	35	61	0.4309
No	---	---			6	9	15		6	14	20	
SKA3 fold change ≥ 1.5			81								93	
Yes	48	6	54	0.0305	---	---			33	27	60	0.0021
No	18	9	27		---	---			7	26	33	
DSN1 fold change ≥ 1.5			81				93					
Yes	26	6	32	0.4309	33	7	40	0.0021	---	---		
No	35	14	49		27	26	53		---	---		

[#], Patients with MSI in either carcinoma or polyp tissues were excluded from the statistical analysis; patients with adenocarcinoma nodules detected in polyp tissues and those with extreme alteration of protein expression were also excluded from the statistical analysis. *, CIN index 0 corresponds to CIN-stable, 1 corresponds to low degree of CIN, 2 corresponds to medium degree of CIN, 3 corresponds to high degree of CIN, and 4 corresponds to ultra-high degree of CIN. [&], Samples with suspected amplification of the *NSE1* gene, the normalization control for genomic qPCR, identified by SNP genotyping array were excluded from statistical analysis.

Supplementary Table S8: Sequences of siRNA oligos

Duplex Catalog Number	Gene Symbol	GENE ID	Gene Accession	GI Number	Sequence
J-018397-05	DSN1	79980	NM_024918	31542256	GUCUAUCAGUGUCGAUUUA
J-018397-06	DSN1	79980	NM_024918	31542256	UGACAUAUCUUGGGUCUUC
J-018397-07	DSN1	79980	NM_024918	31542256	GAACCCACCUGCCAUACAU
J-018397-08	DSN1	79980	NM_024918	31542256	GAUCAGCUCUUGCUUCACU
J-015700-05	SKA3	221150	NM_145061	47419927	GGAAGAGCCCGUAAUUGUA
J-015700-06	SKA3	221150	NM_145061	47419927	GAUCGUACUUCGUUGGUUU
J-015700-07	SKA3	221150	NM_145061	47419927	AAUCCAGGCUCAAUGAUAA
J-015700-08	SKA3	221150	NM_145061	47419927	CAUCGUAUCCCAAGUUCUA

REFERENCES

1. Cox MP, Peterson DA, Biggs PJ. SolexaQA: At-a-glance quality assessment of Illumina second-generation sequencing data. BMC bioinformatics. 2010; 11:485.
2. Langmead B, Trapnell C, Pop M, Salzberg SL. Ultrafast and memory-efficient alignment of short DNA sequences to the human genome. Genome biology. 2009; 10:R25.
3. Trapnell C, Pachter L, Salzberg SL. TopHat: discovering splice junctions with RNA-Seq. Bioinformatics. 2009; 25:1105-1111.
4. Trapnell C, Williams BA, Pertea G, Mortazavi A, Kwan G, van Baren MJ, Salzberg SL, Wold BJ, Pachter L. Transcript assembly and quantification by RNA-Seq reveals unannotated transcripts and isoform switching during cell differentiation. Nature biotechnology. 2010; 28:511-515.
