# Supplementary material for: Over-expression of AURKA, SKA3 and DSN1 contributes to colorectal adenoma to carcinoma progression
Source: Oncotarget. 2016 Jun 13;7(29):45803–18. doi: 10.18632/oncotarget.9960 (PMC5216762; doi:10.18632/oncotarget.9960)
Supplement: Supplementary file 2 [file oncotarget-07-45803-s002.docx]

**Supplementary Table S3: List of genes with differential copy number alterations and progressively deregulated expression.**

| Gene Symbol | Fold Change (adenoma/normal) | Fold Change (carcinoma/normal) | Fold Change (carcinoma/adenoma) |
| --- | --- | --- | --- |
| AAR2 | 1.5039 | 2.4921 | 1.6571 |
| ABHD3 | -1.3524 | -2.7994 | -2.0699 |
| AHCY | 1.5274 | 2.8336 | 1.8552 |
| ANKRD12 | -1.4875 | -2.3219 | -1.5610 |
| ANLN | 1.4571 | 3.6524 | 2.5066 |
| ATP11A | 1.8233 | 3.4255 | 1.8788 |
| ATP2A3 | -1.1096 | -2.6216 | -2.3627 |
| ATP8B1 | -1.4229 | -2.4085 | -1.6927 |
| AURKA | 1.1402 | 2.9226 | 2.5633 |
| BCAS1 | -1.3242 | -3.5311 | -2.6667 |
| BCL2 | -1.4694 | -3.1190 | -2.1226 |
| BRCA2 | 1.1544 | 2.4584 | 2.1297 |
| BZW2 | 1.4309 | 2.4251 | 1.6948 |
| C8orf33 | 1.1236 | 2.3106 | 2.0564 |
| CBX3 | 1.2751 | 2.5189 | 1.9754 |
| CCDC68 | -2.0292 | -3.7397 | -1.8429 |
| CDK5RAP1 | 1.4165 | 2.1580 | 1.5235 |
| CEP250 | 1.4487 | 2.2775 | 1.5720 |
| CKAP2 | 1.4439 | 2.7833 | 1.9276 |
| COL4A1 | 1.0120 | 3.2506 | 3.2121 |
| CPNE1 | 1.5962 | 3.2952 | 2.0643 |
| CTHRC1 | -1.4303 | 5.5936 | 8.0006 |
| DSCC1 | 1.1650 | 3.1204 | 2.6784 |
| DSN1 | 1.2170 | 2.4530 | 2.0156 |
| EIF3B | 1.5366 | 2.5237 | 1.6423 |
| FAM83D | -1.1426 | 2.6501 | 3.0281 |
| FIGNL1 | 1.1746 | 2.5104 | 2.1373 |
| FTSJ2 | 1.3576 | 2.2235 | 1.6378 |
| GINS1 | 1.2027 | 3.4835 | 2.8965 |
| GPT | -1.6637 | -5.5778 | -3.3526 |
| GTF3A | 1.5890 | 3.0715 | 1.9329 |
| LINC00675 | -1.7431 | -2.9876 | -1.7140 |
| LOXL2 | 1.0622 | 3.0631 | 2.8836 |
| LSM5 | 1.2148 | 2.2642 | 1.8639 |

**Supplementary Table S3: List of genes with differential copy number alterations and progressively deregulated expression (Cont’d).**

| Gene Symbol | Fold Change (adenoma/normal) | Fold Change (carcinoma/normal) | Fold Change (carcinoma/adenoma) |
| --- | --- | --- | --- |
| LY6E | 1.1294 | 3.6777 | 3.2564 |
| MIR17HG | 1.3705 | 3.8468 | 2.8069 |
| MOCS3 | 1.1944 | 2.9587 | 2.4771 |
| MRGBP | 1.3979 | 2.8578 | 2.0443 |
| MYBL2 | 1.2343 | 3.7391 | 3.0293 |
| MZT1 | 1.1477 | 2.2326 | 1.9454 |
| NANP | 1.2850 | 2.6565 | 2.0673 |
| NEDD4L | -1.3064 | -2.5711 | -1.9681 |
| NELFCD | 1.4818 | 2.8236 | 1.9056 |
| NFE2L3 | 1.9846 | 4.0215 | 2.0264 |
| PDRG1 | 1.7366 | 3.2749 | 1.8858 |
| PFDN4 | 1.2847 | 2.8695 | 2.2336 |
| PMAIP1 | -1.0230 | 2.6317 | 2.6923 |
| POLR1D | 1.6067 | 2.7828 | 1.7320 |
| PROCR | -1.0532 | 2.4022 | 2.5299 |
| RAE1 | 1.5328 | 2.6276 | 1.7142 |
| RBL1 | 1.0710 | 2.4608 | 2.2976 |
| RFC3 | 1.2548 | 2.8494 | 2.2708 |
| RILP | -1.1015 | -2.3155 | -2.1022 |
| SKA3 | 1.2854 | 2.9557 | 2.2993 |
| SMOX | 2.6878 | 4.7804 | 1.7786 |
| SNHG15 | 1.6533 | 3.4643 | 2.0954 |
| SULF1 | -1.1886 | 4.3281 | 5.1445 |
| TCFL5 | 1.2695 | 2.6508 | 2.0881 |
| TGDS | 1.4451 | 2.2932 | 1.5869 |
| TGIF2 | 1.2420 | 4.0057 | 3.2253 |
| TOMM34 | 1.7404 | 3.4990 | 2.0105 |
| TOX | -1.1770 | -3.5742 | -3.0367 |
| TP53RK | 1.0113 | 2.2596 | 2.2344 |
| TPX2 | 1.4449 | 3.8482 | 2.6633 |
| TRIB3 | 2.8012 | 13.4808 | 4.8125 |
| TTI1 | 1.3349 | 2.4486 | 1.8343 |
| UBE2C | 1.2580 | 4.5948 | 3.6526 |
| ZBTB7C | -1.3394 | -4.6913 | -3.5027 |
